# Outcomes of catheter ablation for ventricular tachycardia in structural heart disease: a meta-analysis and quality appraisal of trials

**DOI:** 10.1093/ehjopen/oeaf171

**Published:** 2025-12-11

**Authors:** Debbie Falconer, Ahmed Salih, Gabriella Captur, Richard J Schilling, Pier D Lambiase, Nikos Papageorgiou, Rui Providencia

**Affiliations:** UCL Institute of Cardiovascular Science, University College London, Gower Street, London WC1E 6BT, UK; School of Medicine, Imperial College London, The Faculty Building, Imperial College London, Exhibition Road, South Kensington, London SW7 2AZ, UK; UCL Institute of Cardiovascular Science, University College London, Gower Street, London WC1E 6BT, UK; Royal Free Hospital, Pond Street, London NW3 2QG, UK; Bart’s Heart Centre, St Bartholomew's Hospital, West Smithfield, London EC1A 7BE, UK; UCL Institute of Cardiovascular Science, University College London, Gower Street, London WC1E 6BT, UK; Bart’s Heart Centre, St Bartholomew's Hospital, West Smithfield, London EC1A 7BE, UK; UCL Institute of Cardiovascular Science, University College London, Gower Street, London WC1E 6BT, UK; Bart’s Heart Centre, St Bartholomew's Hospital, West Smithfield, London EC1A 7BE, UK; Bart’s Heart Centre, St Bartholomew's Hospital, West Smithfield, London EC1A 7BE, UK; Institute of Health Informatics Research, University College London, 222 Euston Road, London NW1 2DA, UK

**Keywords:** arrhythmia, Catheter ablation, Evidence synthesis, Ventricular arrhythmia, Sudden cardiac death

## Abstract

**Aims:**

Catheter ablation (CA) of ventricular tachycardia (VT) in patients with structural heart disease is usually reserved for those with recurrent implantable cardioverter defibrillator (ICD) shocks or intolerant to anti-arrhythmic drugs. This meta-analysis synthesizes available trial evidence on CA for VT to clarify the role of this approach.

**Methods and results:**

MEDLINE, PubMed, EMBASE and Cochrane were searched for randomized controlled trials (RCTs) of patients with structural heart disease allocated to receive either CA or standard treatment. Outcomes of interest were: all-cause and cardiovascular (CV) mortality, VT recurrence, incidence of appropriate ICD therapy, CV hospitalizations and VT storm. Evidence was appraised using the risk of bias tool and the grading of recommendations assessment, development and evaluation (GRADE) approach. Trial-level pairwise meta-analyses were conducted for all outcomes. Reconstructed time-to-event data meta-analysis was also performed for all-cause mortality 13 RCTs (*n* = 1735 patients) were included in the meta-analysis with a follow-up duration of 6–52 months. No significant reduction in all-cause mortality was observed at trial level meta-analysis (risk ratio [RR] 0.87, 95% confidence interval [CI] 0.70–1.08, heterogeneity [I^2^] = 0%), or reconstructed individual patient data meta-analysis [hazard ratio (HR) 0.79, 95%CI 0.57–1.11 at 3 years]. However, our pooled estimates, observed effect size and GRADE assessments suggest a potential mortality reduction in the ablation group. Patients who underwent CA experienced a significant reduction in CV hospitalizations (RR 0.78, 95%CI 0.65–0.94, I^2^ = 41%), VT storm (RR 0.78, 95%CI 0.63–0.97; *I^2^* = 5%), VT recurrence (RR 0.83, 95%CI 0.72–0.95, I^2^ = 21%), and appropriate ICD therapy (RR 0.74, 95%CI 0.61–0.89, I^2^ = 32.5%) compared to control groups.

**Conclusion:**

A potential all-cause mortality reduction by catheter ablation requires further confirmation in a properly powered RCT. No reduction in cardiovascular mortality was found. VT recurrence, CV hospitalizations, VT storm and ICD therapy were all significantly reduced by catheter ablation in patients with structural heart disease.

**Lay summary:**

We examined the effectiveness of catheter ablation (CA) for treating ventricular tachycardia (VT) in patients with structural heart disease, particularly those facing recurrent implantable cardioverter defibrillator shocks or unable to tolerate medications by analysing several randomized controlled trials. The findings suggest that while CA may not significantly reduce overall mortality, it can lead to fewer recurrences of VT and hospitalizations related to cardiovascular problems.

## Graphical Abstract

**Graphical Abstract:**
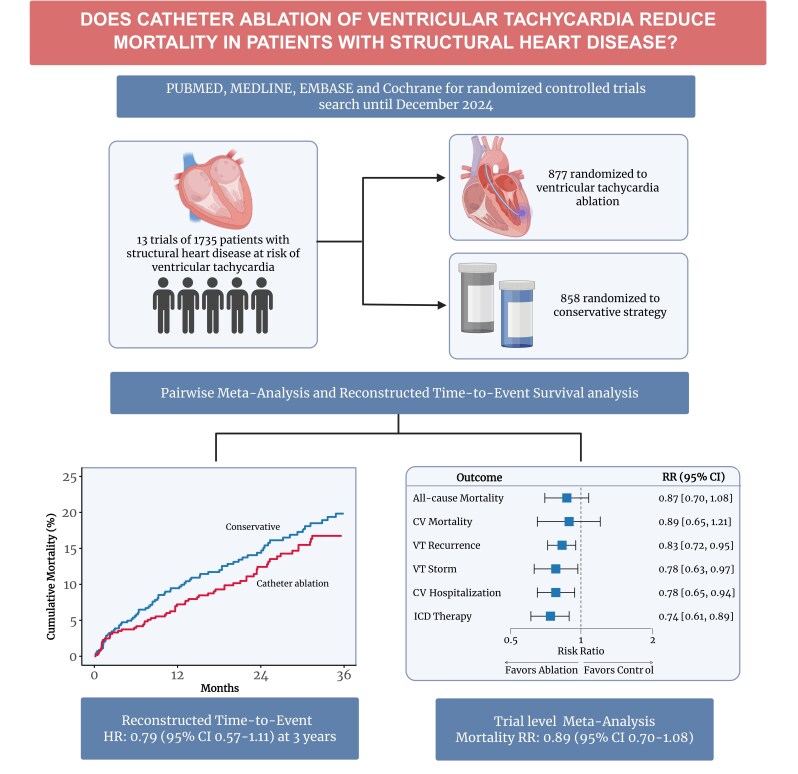

## Introduction

Patients with structural heart disease secondary to cardiomyopathy or ischaemic heart disease (IHD) are at lifelong risk of ventricular tachycardia (VT), necessitating long-term pharmacotherapy to reduce arrhythmia risk, and implantable cardiac defibrillators (ICDs) to prevent sudden cardiac death (SCD).^1^

Current management of VT involves arrhythmia prevention through optimization of heart failure medication and avoidance of exacerbating triggers. ICDs are placed according to international guidelines to treat ventricular arrhythmias and prevent SCD.^2,3^ However, repeated ICD shocks are associated with depression,^4^ post-traumatic stress disorder^5^ and increased mortality.^6^ Evidence of localized myocardial injury following shocks has also been found at autopsy.^7^ Therefore, class I or III anti-arrhythmic drugs (AADs) are usually added if VT persists. However, use of these drugs carries a range of side effects, including hepatotoxicity, pulmonary fibrosis and QT interval prolongation with proarrhythmic consequences.^8^

Decades of development in ablation techniques, equipment and substrate mapping underpin present-day catheter ablation (CA)^9^ which has emerged as an important and effective treatment for VT.^10^ Urgent CA has a class I recommendation to treat electrical storm in the European Society of Cardiology (ESC) guidelines^2^ when medical therapy and ICD re-programming fail. The ESC guidelines^2^ also recognize its importance in preventing VT–CA should be considered in those with recurrent ICD therapies despite beta blocker use (class IIa recommendation; evidence level C), and can be considered alongside ICD implantation to reduce the future shock burden (class IIb; evidence level B). American Heart Association/American College of Cardiology (AHA/ACC) 2017 guidelines adopt a similar position, advising CA for people in whom AADs are ineffective or not tolerated (class I recommendation; evidence level B).^3^

Recent meta-analyses have assessed the efficacy of CA for VT, offering important insights for clinicians.^11–13^ However, two important RCTs with large heterogenous cohorts have since been published– one uniquely focusing on primary prevention and the other comprising the largest CA RCT to date. This meta-analysis therefore aims to comprehensively synthesize the most up-to-date evidence on the efficacy of CA for VT in patients with structural heart disease, analysing the largest available dataset, assessing a wide range of outcomes, and performing detailed subgroup analyses.

## Methods

The meta-analysis was conducted to fulfil the Preferred Reporting Items for Systematic Reviews and Meta-Analysis (PRISMA) criteria on published peer-reviewed journal articles, but also included conference abstracts^14^ (see Supplementary material online, Table  *S*-1). The protocol was prospectively registered on PROSPERO In November 2024 (ID CRD42024619649). The Patient/Intervention/Comparator/Outcomes (PICO) approach was used.^15^ The population of interest included patients with structural heart disease (ischaemic and non-ischaemic) with or at risk of having VT. The intervention of interest was CA. Control groups received new AADs, escalating doses of AADs or no AADs. ICDs were implanted in patients in the intervention and control groups. The primary outcomes of interest were: All-cause and cardiovascular (CV) mortality. Secondary outcomes were VT recurrence, appropriate ICD therapies, VT storm and CV hospitalization. The initial primary outcome was VT recurrence (as stated on the PROSPERO registration), but this was amended during the review process, before data analysis, to reflect more consistent data availability.

### Search strategy

Two reviewers (DF and AS) systematically searched the electronic databases MEDLINE, PUBMED, EMBASE and Cochrane using the following expression: (‘catheter ablation’ OR ‘radiofrequency ablation’) AND (‘ventricular tachycardia’ OR ‘ventricular arrhythmia’) AND (‘structural heart disease’ OR ‘ischaemic heart disease’). The search was limited to studies on adult human subjects published in English-language peer-reviewed journals from 1995 until December 2024. Reference lists of all accessed full-text articles were hand-searched for sources of relevant additional information. The authors of full-text papers and congress abstracts were also contacted by e-mail to retrieve additional information.

### Study selection

**Figure 1:**
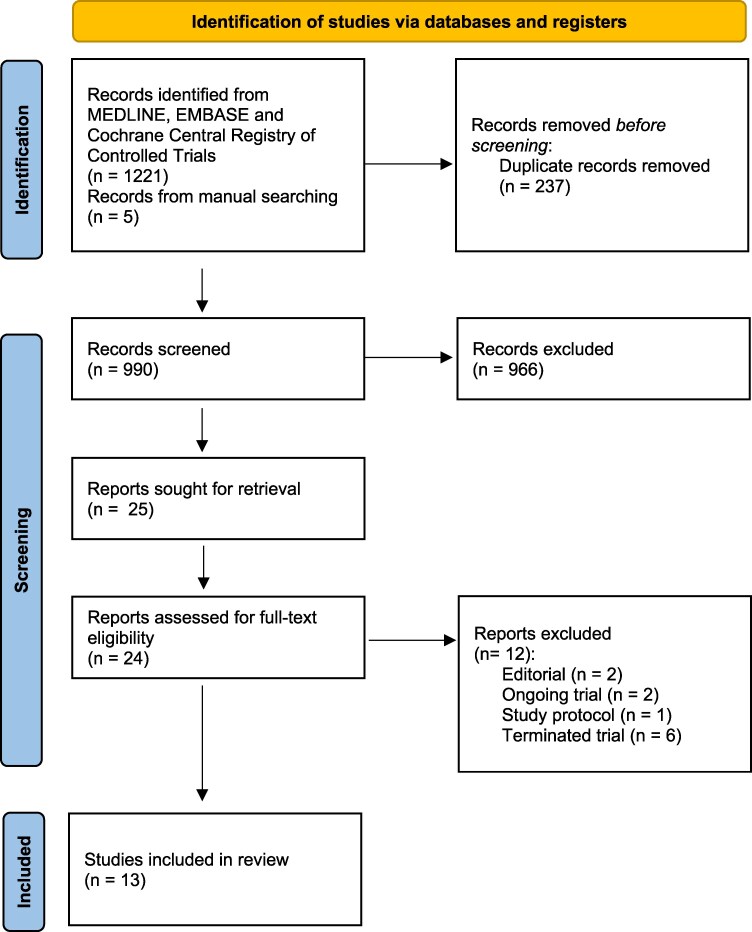
PRISMA flow-chart demonstrating study selection process.

Prospective RCTs published as abstracts or original articles in peer-reviewed scientific journals in English were included. Studies pertaining to the treatment of electrical storm or acute ischaemia, or not reporting outcomes of interest, were excluded. Two reviewers (DF and AS) independently screened all abstracts and titles to identify eligible studies. Full texts were then evaluated. A third author (RP) was consulted in cases of disagreement. Agreement of at least two reviewers was required for decisions regarding inclusion or exclusion of studies. The study selection protocol is provided in *Figure 1*.

### Data extraction

Two authors (DF and AS) independently abstracted trial-level data. Information collected included author, year of publication, interventions, sample size, baseline characteristics, use of AADs, procedural information, outcomes, pertinent past medical history and complications.

### Quality appraisal

Cochrane ‘risk of bias’ tool version 2 was applied by assessing the following domains: randomization, deviation from intended intervention, missing outcome data, measurement of the outcome, selection of reported result, and other bias (e.g. evidence of prospective trial registration). Each study was classified as high, low, or unclear risk of bias by two review authors (MA and RP). Disagreements were resolved by a third author (DF).

The grading of recommendations assessment, development and evaluation (GRADE) approach was taken to assess certainty of outcome evidence.^16^ The GRADE approach appraises the certainty of evidence based on the extent to which one can be confident that an estimate of effect or association reflects the item being assessed. The certainty measure considers within-study risk of bias, directness of the evidence, heterogeneity of the data, precision of effect estimates, and risk of publication bias. The decision to downgrade the certainty of evidence resulted from a consensus between two authors (RP and AS), and a third, if needed (DF).

### Sub-group and sensitivity analyses

To assess the impact of study design on outcomes, the following sub-group analyses were performed:

- type of anti-arrhythmic drug approach
- ablation strategy
- studies recruiting IHD patients only
- secondary prevention studies only
- follow-up duration

Sensitivity analyses were also performed for:

- publication year
- risk of bias
- published manuscripts (excluding abstracts and unpublished data)

These were only performed for conditions fulfilled by at least 2 studies.

Where appropriate to perform subgroup analysis, the median and interquartile range were used to estimate the mean and standard deviation using the formula derived by *Hozo et a*l.^17^

### Data analysis

Trial-level pairwise data were pooled using the Mantel–Haenszel random-effects model. Risk ratios (RR) and 95% confidence intervals (CI) were used as the measure of treatment effect for all outcomes. Visual inspection of contour-enhanced funnel plots^18^ (when at least ten studies were included) was performed to assess for publication bias. Asymmetrical funnel plots were interpreted as indicating the possibility of publication bias. Statistical significance was determined using two-tailed tests, with a *P*-value of <0.05 considered significant. Statistical heterogeneity on each outcome of interest was quantified using the Higgins *I^2^* statistic. The *I^2^* statistic describes the percentage of total variation across studies because of heterogeneity rather than chance. Values of <25%, 25% to 50%, and >50% are by convention classified low, moderate, and high degrees of heterogeneity, respectively. A meta-regression was performed to investigate the effect of the proportion of ischemic cardiomyopathy participants on the outcomes. The analyses were performed using R version 4.3.4, ‘meta’ and ‘metafor’ package.

A reconstructed individual patient data analysis from published Kaplan-Meier (KM) curves was conducted for the primary outcome of all-cause mortality. This approach allowed for more precise and robust estimates by directly incorporating individual-level time-to-event data, which is often limited in trial-level meta-analyses. In this study, the two-stage approach described by Liu et al^19^ was followed to reconstruct individual patient data from published KM curves using the R package ‘IPDfromKM’ (version 0.1.10). KM curves were digitized, raw data coordinates extracted, and individual patient data reconstructed using the modified KM estimation algorithm (modified-iKM) from Guyot et al.^20^ The quality of the reconstruction was validated by comparing at-risk tables, hazard ratios (HRs), and visually inspecting the KM curves.

The individual patient data from all studies were pooled into a single dataset, and survival curves were generated using the R package ‘survival’. A Cox-based shared-frailty model, treating trial as a random effect, was used to estimate pooled HRs and 95% confidence intervals (CIs). The primary analysis was conducted at a 3-year follow-up period, as this was the point at which at least half of the studies reported data. The proportional hazards assumption was verified using the Grambsch–Therneau test and visually by plotting the Schoenfeld residuals. Flexible parametric survival models and landmark analysis were performed if proportional hazards assumptions were violated. A sensitivity analysis was conducted by comparing hazard ratios at the trial level meta-analysis.

The Number Needed to Treat (NNT) or Number Needed to Harm (NNH), and respective 95% confidence intervals were calculated,^21,22^ where applicable. These were estimated as the reciprocal of the absolute risk difference for the particular outcome between treated subjects and the control or placebo group, i.e.:


$$NNT=\;\frac{1}{Absolute\;Risk_{Control\;Group}-Absolute\;Risk_{Treatment\;Group}}$$

## Results

The systematic review identified 13 RCTs,^23–35^ including one abstract^23^ and one unpublished study,^35^ after screening and exclusion (*Figure 1*)(*n* = 1735 patients, 94.4% male). Reasons for exclusion are presented in Supplementary material online, Table  *S*-2. Two ongoing RCTs were identified (see Supplementary material online, Table  *S*-3).

Baseline characteristics are summarized in *Table 1*. The mean follow-up duration in Epstein et al. and CALYPSO was six months, whilst all other studies performed longer follow-up of 13.2–52 months. Ten RCTs included patients with IHD only, whereas three studies recruited patients with IHD and NICM.^23,29,34^ PREVENTIVE-VT recruited patients having ICDs for primary prevention only. PAUSE-SCD recruited patients who met both primary and secondary prevention criteria, though all other studies investigated CA in the context of secondary prevention. All studies except Epstein implanted ICDs in 100% of patients (either prior to or during the study). One study, ERASE-VT^33^ remains unpublished, meaning limited data were available. However, available information pertaining to study protocol and outcomes was extracted from a prior meta-analysis^11^ which had access to patient-level data.

**Table 1:** Baseline characteristics

Author, year	Acronym	RCT comparison	Population	Primary/ secondary/mixed	Single vs. Multicentre	*n*	ICD *in situ*/inserted during study *n*(%)	Amiodarone at enrolment *n* (%)	Beta-blockers *n* (%)	Age (mean ± SD or median(IQR)	Male %	Aetiology	LVEF (%). Mean ± SD or median (IQR)
Epstein (1998)^23^	–	Abl vs. AADs	VT with structural heart disease	Secondary	Multi	Ablation 73 Control 32	51 (70) 24 (75)			62.5 ± 19.8 66.7 ± 19.8	92 84	Ischaemic 83% Ischaemic 91%	31 ± 13 29 ± 12
Reddy (2007)^24^	SMASH-VT	Abl and ICD vs. ICD alone, no AADs	IHD with unstable VT/VF or after one ICD shock	Secondary	Multi	Ablation 64 Control 64	64 (100) 64 (100)	0 (0) 0 (0)	60 (94) 63 (98)	67 ± 9 66 ± 10	92 81	Ischaemic 100%	30.7 ± 9.5 32.9 ± 8.5
Kuck (2010)^25^	VTACH	Abl and ICD vs. ICD alone	IHD with stable VT and EF <50%	Secondary	Multi	Ablation 52 Control 55	52 (100) 55 (100)	18 (35) 19 (35)	39 (75) 41 (75)	67.7 ± 8.3 64.4 ± 8.2	96 91	Ischaemic 100%	34.0 ± 9.6 34.1 ± 8.8
Al-Khatib (2014)^26^	CALYPSO	Abl vs. AADs^a^, no prior AAD	IHD with ICD and 1 shock or 3x ATP	Secondary	Multi	Ablation 13 Control 14	13 (100) 14 (100)	0 (0) 0 (0)	13 (100) 12 (86)	64 (44–81) 65 (43–81)	100 86	Ischaemic 100%	25 (15–65) 23 (10–45)
Sapp (2016)^27^	VANISH	Abl vs. escalating AADs	IHD and device treatment for VT- with ICD and AAD	Secondary	Multi	Ablation 132 Control 127	132 (100) 127 (100)	85 (64.4) 84 (66.1)	124 (93.9) 122 (96.1)	67.0 ± 8.6 70.3 ± 7.3	93 93	Ischaemic 100%	31.1 ± 10.4 31.2 ± 10.7
Kuck (2017)^28^	SMS	Abl and ICD vs. ICD alone	IHD with unstable VT EF <40%	Secondary	Multi	Ablation 54 Control 57	54 (100) 57 (100)	16 (30) 20 (35)	49 (91) 52 (91)	68 ± 8 66 ± 8	87 81	Ischaemic 100%	32.0 ± 6.9 30.4 ± 7.3
NCT 01182389^33^	ERASE-VT	Abl vs. AADs	IHD and VT with ICD	Secondary	Multi	Ablation 26 Control 25				69	84	Ischaemic 100%	31.2
Willems (2020)^35^	BERLIN-VT	Abl + ICD vs. ICD ± deferred ablation	IHD, LVEF 30–50% and documented VT	Secondary	Multi	Ablation 76 Control 83	76 (100) 83 (100)	31 (40.8) 22 (26.5)	58 (76.3) 59 (71.1)	66 ± 10 66 ± 9	88.2 86.7	Ischaemic 100%	41 ± 6 41 ± 6
Tung (2022)^34^	PAUSE-SCD	Abl + ICD vs. AADs + ICD	IHD/NI-DCM/ARVC with ICD indication	Mixed	Multi	Ablation 60 Control 61	60 (100) 61 (100)	16 (28.6) 20 (32.8)	47 (78.3) 53 (86.9)	51 (45.5–65) 57 (47–63)	73.3 88.5	Ischaemic 33.3% Ischaemic 36.1%	41 (31–60) 40 (30–48)
Della Bella (2022)^29^	PARTITA	Abl vs. AADs	NI-DCM/IHD post 1 ICD shock	Secondary	Multi	Ablation 23 Control 24	23 (100) 24 (100)	1 (5) 4 (21)	23 (100) 24 (100)	71.2 ± 8.1 65.6 ± 9.6	83 88	Ischaemic 87% Ischaemic 75%	31.9 ± 9.0 32.4 ± 8.3
Arenal (2022)^30^	SURVIVE-VT	Abl vs. AADs^b^	IHD and ICD with symptomatic VT (shock or syncope)	Secondary	Multi	Ablation 71 Control 73	71 (100) 73 (100)	0 (0) 0 (0)	69 (97.2) 62 (86.1)	70 (63–75) 71 (64–76)	98.6 93.2	Ischaemic 100%	35 (26–41) 33 (25–40)
Žižek (2024)^32^	PREVENTIVE-VT	Abl + ICD vs. ICD alone	EF < 40% and scar related to CTO- no previous VT/VF	Primary	Multi	Ablation 30 Control 30	30 (100) 30 (100)	0 (0) 0 (0)	29 (96.7) 29 (96.7)	65 (57–63) 71 (66–76)	96.7 86.7	Ischaemic 100%	37 (32.5–41.5) 34 (30–38)
Sapp (2024)^31^	VANISH-2	Abl vs. AADs (+ICD)	IHD and VT whilst off AADs	Secondary	Multi	Ablation 203 Control 213	203 (100) 213 (100)	0 (0) 0 (0)		67.7 ± 8.6 68.4 ± 8.0	95.1 92.5	Ischaemic 100%	34 ± 11 34.3 ± 10.3

**Abbreviations**: AAD: antiarrhythmic drug; Abl: ablation; ARVC: arrhythmogenic cardiomyopathy; ATP: anti-tachycardia pacing; CTO chronic total occlusion; EF: ejection fraction ICD: implantable cardiac defibrillator; IHD: ischaemic heart disease; IQR: interquartile range; NI-DCM: non-ischaemic dilated cardiomyopathy; RCT: randomized-controlled trial; SD: standard deviation; VT: ventricular tachycardia; VF: ventricular fibrillation Boxes have been left blank where information not supplied.

^a^All on one of amiodarone, mexiletine, ranolazine, dofelitide.

^b^All on amiodarone alone/amiodarone and beta blockers/sotalol and beta blockers.

Four studies offered endo-epicardial procedures,^26,31–33,^ whilst all others performed endocardial procedures only. CALYPSO (*n* = 27) and PREVENTIVE-VT (*n* = 60) performed endocardial procedures in the first instance, and epicardial if the initial ablation was unsuccessful. PAUSE-SCD (*n* = 133) performed epicardial ablation in 55% of cases, operators being encouraged (but not mandated) to do so in NICM and VANISH-2 performed endocardial ablation, and epicardial ablation if VT remained inducible. In three trials (SMASH-VT,^24^ PARTITA,^29^ & PREVENTIVE-VT,^32^) no class I or III AADs were used in either arm at baseline or as part of study treatment. Details on study interventions are provided in *Table 2*.

**Table 2:** Intervention details

Study	Index arrhythmia	Ablation strategy	Mapping system	Follow-up duration, months (mean ± SD unless stated)	Anti-Arrhythmic Therapy
Epstein (1998)^23^	VT			6	
Reddy (2007)^24^	VF; VT; syncope and inducible VT; ICD therapy for VT/VF	Endocardial 100%	CARTO (Biosense Webster, Inc., Diamond Bar, CA, USA)	22.5 ± 5.5	No AADs; control arm received ICD implantation
Kuck (2010)^25^	VT with no syncope/arrest	Endocardial 100%	CARTO (Biosense Webster, Inc., Diamond Bar, CA, USA) OR Ensite (St Jude Medical, St Paul, MN, USA)	22.5 ± 9	Both arms β-blockers and amiodarone
Al-Khatib (2015)^26^	VT with 1 shock/3 ATP	Endocardial preferred, epicardial if unsuccessful	Discretion of treating physician	6	Control arm only- First-line therapy: amiodarone and sotalol; Second-line therapy: mexiletine, ranolazine and dofetilide. β-Blockers
Sapp (2016)^27^	VT with 1 shock/3 ATP; suspected VT below detection zone	Endocardial 100%		27.9 ± 17.1	Both arms: Amiodarone or another Class I or Class III AAD at enrolment; Continued in the ablation arm and escalated in controls.
Kuck (2017)^28^	Spontaneous unstable VT; syncope with inducible VT; cardiac arrest with VT	Endocardial 100%	CARTO (Biosense Webster, Inc., Diamond Bar, CA, USA) OR Ensite (St Jude Medical, St Paul, MN, USA)	27.6 ± 13.2	Both arms: Pharmacological rhythm control, specifically with amiodarone
ERASE-VT^33^				15	Pharmacological rhythm control, although no changes were made subsequent to enrolment
Willems (2020)^35^	Sustained VT			13.2 ± 9.5	AADs in both arms in in 32.5% to 40.8%, mainly amiodarone.
Tung (2022)^34^	Stable VT; VT with syncope or cardiac arrest; inducible VT	Endocardial 100% Epicardial 55%	Ensite Velocity, Abott, IL	Median 31 (IQR 20.1–40)	Control group: AADs left to the discretion of the treating physician
Della Bella (2022)^29^	Appropriate shock on ICD inserted for primary or secondary prevention	Endocardial 100% Epicardial if required	CARTO (Biosense Webster, Inc., Diamond Bar, CA, USA) OR Ensite (St Jude Medical, St Paul, MN, USA)	Median 28.8 (IQR 16.8–52.8)	No AADs; Exclusion criteria if used, except for amiodarone for AF.
Arenal (2022)^30^	Following appropriate shock for any VT	Endocardial 100%		Median 23.5	Only in the AAD group: Amiodarone + β-blockers, amiodarone alone, or sotalol ± β-blockers
Žižek (2024)^32^	Primary prevention- no documented VT/VF	Endocardial 100% (epicardial for repeat procedure if needed)	CARTO (Biosense Webster, Inc., Invine, CA, USA)	44.7 ± 20.7	No AADs at baseline; Avoided if possible during the study.
Sapp (2025)^31^	VT storm; 1x shock; 3x ATP (1 symptomatic); sustained VT	Endocardial, epicardial if VT remains inducible		Median 52	Control group received AADs with either sotalol or amiodarone.

Abbreviations as per *Table 1*. Boxes have been left blank where information not supplied.

### Quality of included evidence

The risk of bias (ROB) assessment is presented in Supplementary material online, Figure  *S*-1. Epstein *et al.*^11^ was only available as an abstract, and ERASE-VT remains unpublished, limiting a full ROB assessment. Incomplete outcome data (domain 3) and selective reporting (domain 5) were consistently low risk across all studies.

All trials were open-label due to the impracticality of masking treatment allocation for patients and operators, resulting in the outcome ‘some concerns’ for most studies for domain 2 (deviations from intended interventions). This warrants caution when interpreting more subjective outcomes such as cardiovascular hospitalizations and cardiovascular mortality. However, lack of blinding should not impact outcome assessment of objective metrics such as all-cause mortality or device therapy. SURVIVE-VT was classified as high risk in domain 2 due to the high crossover rate between rial arms (>20%).

The PARTITA trial was classified as having ‘some concerns’ in domain 1 (randomization) owing to baseline differences between the two groups (see Supplementary material online, Table  *S*-4).^29^ Studies for which the randomization process was not clearly described were also classified as having ‘some concerns’ for domain 1. Studies in which the outcome reporting was not clearly described (e.g. detailing if trial outcome adjudicators were blinded to intervention) were deemed ‘some concerns’ for domain 4 (measurement of outcomes).

Heterogeneity was low for outcomes except VT recurrence, appropriate ICD therapy and CV hospitalization, where it was considered moderate.

Certainty of evidence was considered moderate or low for most endpoints. This was driven mainly by imprecision (broad confidence intervals in the effect estimates) and performance bias (i.e. lack of blinding) for subjective outcomes (cardiovascular mortality and cardiovascular hospitalizations) (Summary of findings table–Supplementary material online, table  *S*-5).

### Efficacy outcomes

Data on procedural outcomes are summarized in *Table 3*.

**Table 3:** Procedural outcomes

Study	Primary endpoint of trial (composite if multiple)	Group	VT recurrence *n* (%)	VT Storm *n* (%)	All-cause Mortality *n* (%)	Cardiovascular hospitalization *n* (%)	Cardiovascular mortality *n* (%)	Appropriate ICD therapy *n* (%)	Appropriate shocks *n* (%)	Appropriate ATP *n*(%)
Epstein (1998)^23^	VT recurrence	Ablation Control	36 (49) 24 (75) *P* = 0.0004							
Reddy (2007)^24^	Freedom from shock/ATP	Ablation Control		4 (6) 12 (19) HR 0.3 (0.09–1) *P* = 0.06	6 (9) 11 (17) HR 0.59 (0.22–1.59) *P* = 0.29		3 (5) 7 (11)	8 (12) 21 (33) HR 0.35 (0.15–0.78) *P* = 0.007	6 (9) 20 (31) HR 0.27 (0.11–0.67) *P* = 0.003	
Kuck (2010)^25^	Time to recurrence of sustained VT/VF	Ablation Control	28 (53.6) 39 (71.2) HR 0.61 (0.38–1.01*) P* = 0.051	13 (25) 17 (30.3) HR 0.73 (0.36–1.5) *P* = 0.395	4 (8.5) 5 (8.6) HR 1.32 (0.35–4.94) *P* = 0.677	17 (32.6) 30 (54.6) HR 0.55 (0.3–0.99) *P* = 0.044		26 (50) 38 (69.1) *P* = 0.051	14 (26.9) 26 (47.3) *P* = 0.045	
Al-Khatib (2015)^26^	Feasibility of ablation as first-line treatment	Ablation Control	8 (62) 6 (43)		2 (15) 2 (14)	5 (46) 7 (50)				
Sapp (2016)^27^	All-cause mortality, VT storm, appropriate shock	Ablation Control		38 (28.8) 46 (36.2) HR 0.74 (0.48–1.14) *P* = 0.17	36 (27.3) 35 (27.6) HR 0.96 (0.6–1.53) *P* = 0.86	33 (25) 39 (30.7) HR 0.76 (0.48–1.21) *P* = 0.25	24 (18.1) 26 (20.4)		56 (42.4) 54 (42.5) HR 0.97 (0.66–1.4) *P* = 0.85	84 (63.6) 79 (62.2) HR 0.97 (0.71–1.32) *P* = 0.83
Kuck (2017)^28^	Time to recurrence of VT/VF	Ablation Control	25 (46.3)^a^ 26 (45.6)^a^ HR 0.95 (0.55–1.64*) P* = 0.84	4 (7.4) 7 (12.2) HR 0.6 (0.18–2.06*) P* = 0.42	9 (16.7) 11 (19.3) HR 0.82 (0.34–1.97) *P* = 0.65	21 (38.8) 25 (43.9)	2 (3.7) 2 (3.5)	20 (37.0) 24 (42.1) HR 0.81 (0.45–1.47) *P* = 0.49	8 (14.8) 14 (24.6) HR 0.55 (0.23–1.32) *P* = 0.18	
ERASE-VT^33^		Ablation Control	10 (38.5) 14 (56.0)		2 (7.7) 4 (16)					
Willems (2020)^35^	All-cause mortality, hospitalization for VT/VF or HF	Ablation Control	29 (39.7)^a^ 40 (48.2)^a^ HR 0.62 (0.38–1.0) *P* = 0.05		6 (7.9) 2 (2.4) HR 2.97 (0.6–14.7) *P* = 0.18	25 (32.9) 26 (31.3) HR 1.03 (0.59–1.78) *P* = 0.92	1 (1.3) 2 (2.4)	25 (34.2) 39 (47) HR 0.55 (0.33–−0.91) *P* = 0.02	13 (17.8) 18 (21.7) HR 0.7 (0.34–1.44) *P* = 0.34	25 (34.2) 38 (45.8) HR 0.57 (0.34–0.95) *P* = 0.03
Tung (2022)^34^	Recurrent VT, hospitalization, death	Ablation Control	19 (31.7) 31 (50.8) HR 0.51 (0.29–0.9) *P* = 0.02		5 (8.3) 4 (6.6) HR 1.4 (0.38–5.22) *P* = 0.62	17 (28.3) 20 (32.8) HR 0.82 (0.43–1.56) *P* = 0.55	2 (3.3) 3 (4.9)		6 (10.0) 15 (24.6) *P* = 0.03	10 (16.7) 20 (32.8) *P* = 0.04
Della Bella (2022)^29^	All-cause mortality, HF hospitalization	Ablation Control	7 (30.4) 12 (50) *P* = 0.434	0 (0) 2 (8.3) *P* = 0.28	0 (0) 8 (33.3) *P* = 0.004	1 (4.3)^b^ 4 (16.7)^b^ *P* = 0.159	0 (0) 3 (12.5) *P* = 0.087		2 (8.7) 10 (41.7) *P* = 0.039	7 (30.4) 11 (45.8) *P* = 0.639
Arenal (2022)^30^	CV death, appropriate ICD shock, HF hospitalization or severe treatment complication	Ablation Control	19 (26.8) 21 (28.8) HR 0.79 (0.43–1.49) *P* = 0.417	2 (2.8) 5 (6.8) HR 0.38 (0.07–1.98) *P* = 0.252	3 (4.2) 4 (5.5) HR 0.69 (0.15–3.08) *P* = 0.624	13 (18.3) 27 (37.0) HR 0.42 (0.22–0.82) *P* = 0.011	3 (4.2) 3 (4.1) HR 0.923 (0.19–4.61) *P* = 0.929	18 (25.4) 16 (21.9) HR 1.02 (0.52–2.01) *P* = 0.950	12 (16.9) 13 (17.8) HR 0.88 (0.4–1.93) *P* = 0.749	8 (11.4) 12 (16.4) HR 0.54 (0.22–1.34) *P* = 0.186
Žižek (2024)^32^	ICD therapy, hospitalization for VT/VF	Ablation Control		0 (0) 6 (20) *P* = 0.01	8 (26.7) 12 (40) HR 0.55 (0.22–1.37) *P* = 0.194	4 (13.3) 16 (53.3) HR 0.21 (0.07–0.63 *P* = 0.002	4 (13.3) 8 (26.7) HR 0.41 (0.12–1.38) *P* = 0.139	5 (16.7) 12 (40) HR 0.37 (0.13–1.05) *P* = 0.051	5 (16.7) 10 (33.4) *P* = 0.136	
Sapp (2025)^31^	All cause death; VT storm, appropriate shock; sustained VT below detection range	Ablation Control	115 (56.7) 125 (58.7) HR 0.94 (0.73–1.21)	44 (21.7) 50 (23.5) HR 0.95 (0.63–1.42)	45 (22.2) 54 (24.4) HR 0.84 (0.56–1.24)	103 (50.7) 114 (53.4) HR 0.95 (0.79–1.14)	29 (14.3) 25 (11.7) HR 1.23 (0.72–2.10)		60 (29.6) 81 (38) HR 0.75 (0.53–1.04)	96 (47.3) 103 (48.4) HR 0.98 (0.75–1.30)

Abbreviations as per *Table 1*. Boxes have been left blank where information not supplied. Where available, hazard ratios and 95% confidence intervals have been included.

^a^VT or VF.

^b^HF hospitalization only reported.

### All-cause mortality

**Figure 2:**
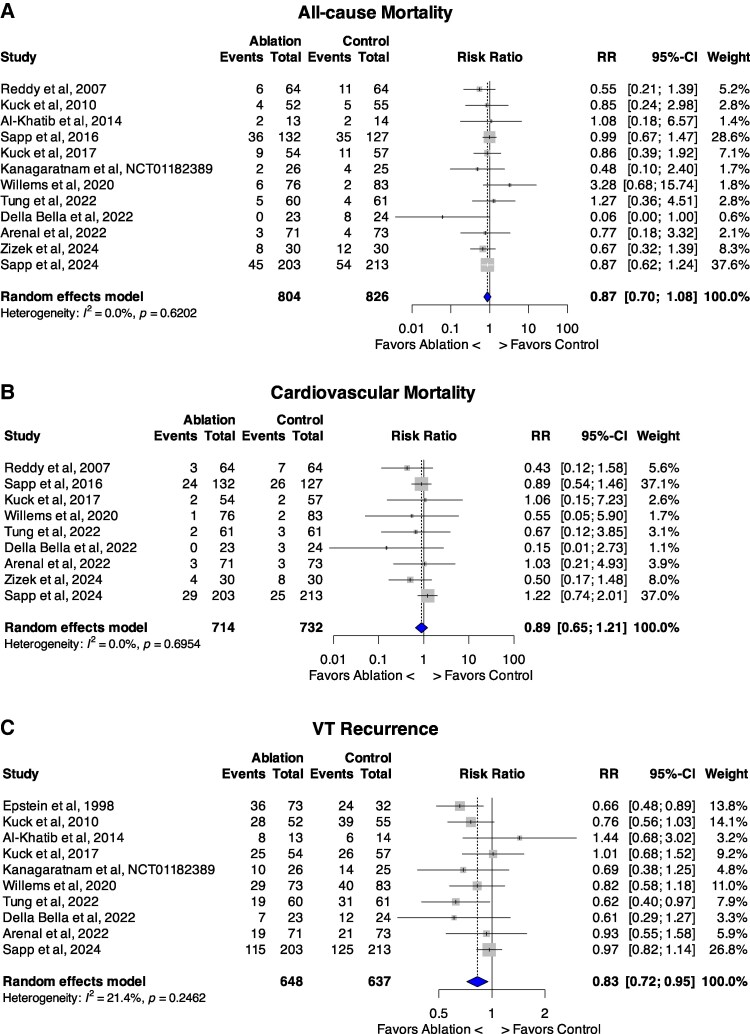
Forest plots of trial-level meta-analysis comparing catheter ablation therapy vs. control for: a, all-cause mortality. B, Cardiovascular mortality. C, VT recurrence. Abbreviation: VT, Ventricular Tachycardia; CI, confidence interval; RR, risk ratio.

12 RCTs reported on call-cause mortality during follow up^24–35^ (*n* = 1630). At trial-level analysis, no significant prognostic benefit was seen following CA (*Figure 2A*). 126 patients in the ablation group died compared with 152 in the control group with low heterogeneity between studies (15.7% vs. 18.4%; RR 0.87, 95%CI 0.70–1.08; *P* = 0.20; *I^2^* = 0%).

Funnel plots excluded publication bias (see Supplementary material online, Figure  *S*-2).

**Figure 3:**
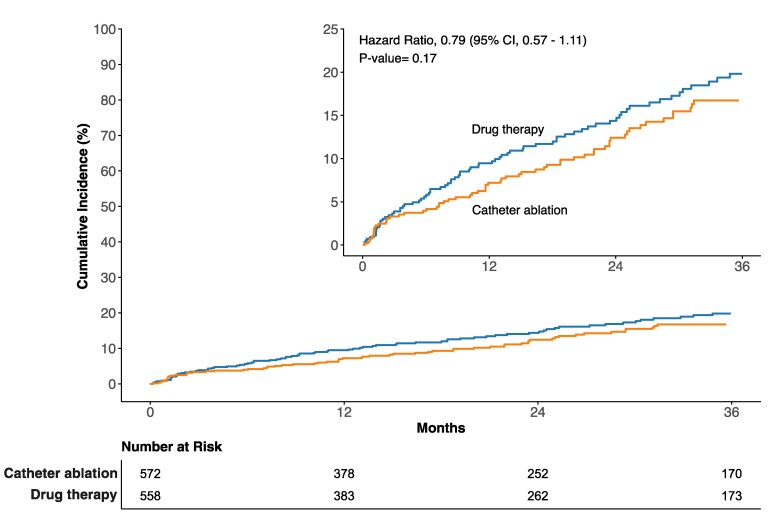
Reconstructed all-cause mortality cumulative incidence curves for individual patient data comparing catheter ablation vs. drug therapy. Individual patient data (IPD) were available for the following studies and were incorporated into the construction of the incidence curve: BERLIN-VT,^35^ PARTITA,^29^ PAUSE-SCD,^34^ SMASH-VT,^24^ VANISH^27^ and VANISH-2.^31^ Abbreviation: CI, confidence interval.

To incorporate time-to-event data, published KM curves from six studies (BERLIN-VT,^35^ PARTITA,^29^ PAUSE-SCD,^34^ SMASH-VT,^24^ VANISH^27^ and VANISH-2^31^) were pooled together using a reconstructed individual patient data analysis (*n* = 1130, 558 CA group, 572 standard therapy group). The reconstructed cumulative incidence curves for each trial (see Supplementary material online, *F*igure  *S*-3) were compared with the original curves for each study. At the prespecified follow-up endpoint of 3-years, a comparable estimate was obtained, with non-significant reduction of mortality in the ablation group (HR 0.79, 95%CI: 0.57–1.11, *P* = 0.17 (*Figure 3*). Significant heterogeneity was found (*P* = 0.003). Similar results were found when analysing at 1- and 2-year follow-up (see Supplementary material online, Table  *S*-6).

There was no visual evidence of a violation of the proportional hazards assumption. The Schoenfeld residuals are shown in Supplementary material online, Figure  *S*-4, and the Grambsch-Therneau test for time-invariant effects had *P*-value of 0.75. Similar results were observed when pooling the hazard ratio at trial level (see Supplementary material online, Figure  *S*-5). The reconstructed time-to-event analysis for trials of IHD only is shown in Supplementary material online, Figure  *S*-6.

### Cardiovascular mortality

Nine studies reported on CV mortality during follow up (*n* = 1446)^24,25,27–32,34^ which occurred in 68 patients in the ablation group compared with 79 in the control group with low heterogeneity between studies (9.5% vs. 10.8%; RR 0.89, 95%CI 0.65–1.21; *P* = 0.46; *I^2^* = 0%; NNT = 78.8) (*Figure 2B*).

### VT recurrence

In ten studies (*n* = 1285)^23,25,26,28–35^ VT recurred in 296 patients in the ablation group compared with 338 in the controls, with low heterogeneity between studies [45.7% vs. 53.1%; RR 0.83, 95%CI 0.72–0.95; *P* = 0.007; *I^2^* = 21.4%; NNT = 13.6, (95%CI 7.8–51.8) patients to prevent one relapse] (*Figure 2C*). Funnel plots excluded publication bias (see Supplementary material online, Figure  *S*-7).

### VT storm

**Figure 4:**
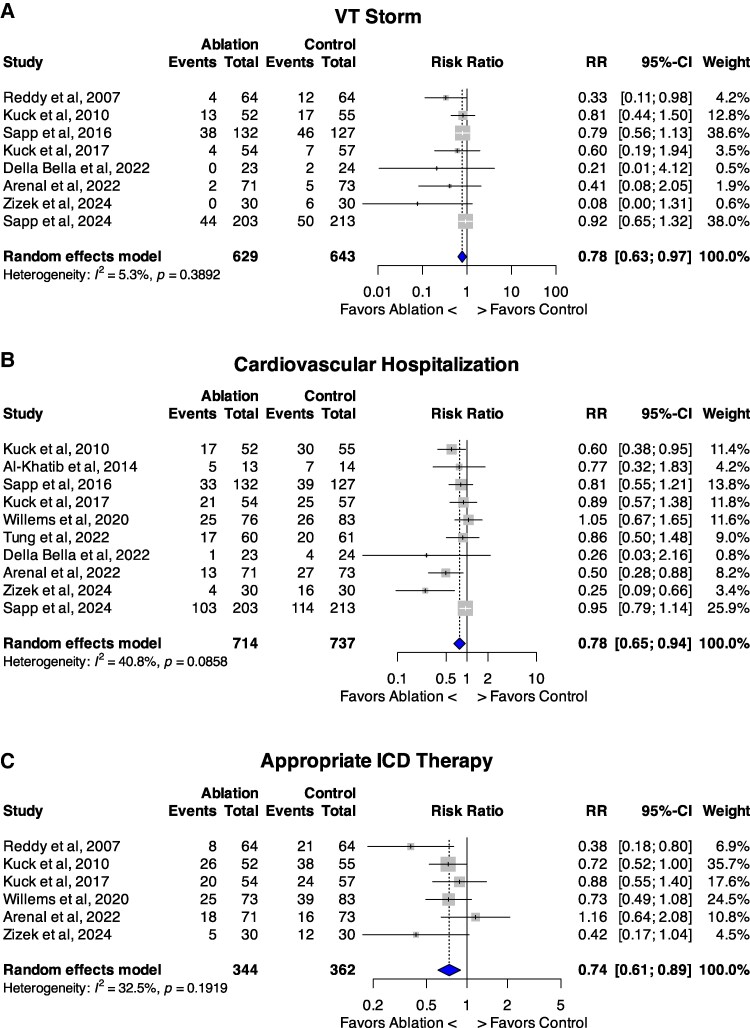
Forest plots comparing catheter ablation therapy vs. control for three clinical outcomes. A, VT Storm. B, Cardiovascular hospitalization. C, Appropriate ICD therapy. Abbreviations: VT, ventricular tachycardia. ICD; implantable cardioverter defibrillator; RR, risk ratio.

Eight studies reported on incidence of VT storm (*n* = 1272)^24,25,27–32^ (*Figure 4A*) which occurred in 105 patients in the ablation group compared with 145 in the control group, with low heterogeneity between studies [17.5% vs. 22.7%; RR 0.78, 95%CI 0.63–0.97; *P* = 0.026; *I^2^* = 5%; NNT = 17.9 (95%CI 10.0–82.7) patients to prevent one VT storm].

### Cardiovascular hospitalizations

CV hospitalization was reported in ten studies (*n* = 1451)^25–32,34,35^ (*Figure 4B*). There was a significant reduction in the ablation group with 239 events, compared with 308 in the control group but with moderate heterogeneity between studies [33.5% vs. 41.8%; RR 0.78, 95%CI 0.65–0.94; *P* = 0.01; *I^2^* = 41%; NNT = 12.0 (95% CI 7.5–29.8) patients to prevent one CV hospitalization]. Funnel plots excluded publication bias (see Supplementary material online, Figure  *S*-8).

### Appropriate ICD therapies

Six studies reported on incidence of appropriate ICD therapies (both shocks and antitachycardia pacing)(*n* = 706).^24,25,28,30,32,35^ There was a significant reduction in therapies: 102 in the ablation group compared with 150 in the control group [29.7% vs. 41.4%; RR 0.74, 95%CI 0.61–0.89; *P* = 0.02, *I^2^* = 32.5%; NNT = 8.5 (95% CI 5.3–20.9) patients to prevent one ICD therapy] (*Figure 4C*).

Ten studies reported on the incidence of appropriate ICD shocks only (*n* = 1549).^24,25,27–32,34,35^ There was a significant reduction in shocks – 182 in the ablation group compared with 261 in the control group [37.3% vs. 43.5%; RR 0.67, 95%CI 0.52–0.86; *P* = 0.002; *I^2^* = 44%; NNT = 10.8 (95%CI 7.3–20.8)] (see Supplementary material online, Figure  *S*-9). There was, however, moderate heterogeneity of 44%.

### Summary of main findings

The pooled estimates hint at a potential mortality reduction effect of catheter ablation, which requires further confirmation in a large and properly powered RCT. No reduction in cardiovascular mortality was found. There was a significant reduction in VT recurrence, VT storm, cardiovascular hospitalizations and ICD therapies.

### Sub-group and sensitivity analyses

Subgroup analyses of solely IHD or secondary prevention studies are shown in Supplementary material online, Table  *S*-7 and *S8*. A separate analysis was conducted of the only trials available as full peer-reviewed publications, excluding Epstein et al and ERASE-VT (see Supplementary material online, Table  *S*-9).^23,33^ No subgroup data of NICM were available from mixed studies, so no subgroup analysis was possible.

There was a trend towards a more pronounced reduction in ICD therapies in lower quality RCTs following CA (*P* = 0.052) and a significantly greater reduction in CV hospitalization in studies performing endocardial ablation only (*P* = 0.02). There was also a significantly larger reduction in electrical storm, CV hospitalization, CV mortality, appropriate ICD therapy, and appropriate ICD shocks following CA in studies with no AAD use (*P* < 0.01) (see Supplementary material online, Table  *S*-10–14). Furthermore, although no significant subgroup differences were observed for all-cause or cardiovascular mortality (*P* = 0.23 and *P* = 0.25, respectively), pooling the three studies without AAD use (SMASH-VT, PARTITA, and PREVENTIVE-VT) revealed a significant reduction in both outcomes: RR 0.56, 95% CI 0.32–0.99 for all-cause mortality, and RR 0.43, 95% CI 0.19–0.96 for cardiovascular mortality.

Meta-regression was used to assess the variability across studies by the proportion of participants with ischemic cardiomyopathy, (see Supplementary material online, Table  *S*-15) and showed a significant effect on VT recurrence but no other outcome. Meta-regression assessing variability by proportion of male patients and by age showed no significant effect on any outcome (see Supplementary material online, Tables  *S*-16 and *S*-17). A leave-one-out sensitivity analysis was conducted by sequentially excluding one study at a time and re-fitting the model of the primary and secondary outcomes. The resulting pooled estimates are shown in Supplementary material online, Figures  *S*-10 and *S*-11.

Detailed information on ICD programming and complications for all trials is presented in Supplementary material online, Tables  *S*-18 and *S*-19. Supplementary material online, Table  *S*-20 provides a comprehensive comparison of this systematic review with other related publications from recent years.

## Discussion

This meta-analysis provides evidence of a significant reduction in VT recurrence, VT storm, CV hospitalization and appropriate ICD therapies following CA in patients with structural heart disease compared with standard therapy. There was no significant reduction in all-cause or cardiovascular mortality at the trial-level data. However, reconstructed KM curves show a trend towards improved all-cause mortality following ablation, with separation of the curves seen as early as one month post-procedure.

The consistent separation of curves hints at a possible mortality benefit. A larger trial would be required to confirm these observations: detection of an absolute 2.5% mortality difference with 80% power at a 0.05 statistical significance would require recruitment of over 7000 patients (3584 in each treatment group) before accounting for potential losses due to follow-up issues or patients not receiving the allocated intervention. Though such vast numbers have been recruited by drug-based trials, they will be more difficult to achieve for an ablation study.

Reconstructing individual patient data from published KM curves has become an increasingly popular method to overcome limitations inherent in conventional trial-level meta-analyses, such as handling censoring and varying follow-up durations. This approach allows for the direct incorporation of individual-level time-to-event data, leading to more precise estimates. Several studies have demonstrated the high reproducibility of reconstructed individual patient data meta-analyses to closely approximate results obtained from original datasets.^36^ However, it is important to interpret these findings with caution. Reconstructed patient data cannot completely replicate original individual-level data, which offers a more comprehensive understanding of participants’ characteristics to explain study heterogeneity. Albeit with a comparable effect estimate (HR 0.79, 95%CI 0.57–1.11, *P* = 0.17), our findings differ slightly from a recently published meta-analysis from Reddy et al^11^ in which the all-cause mortality benefit reached statistical significance (HR 0.73, 95% CI 0.53–1, *P* = 0.047). Notably, Reddy et al. restricted their analysis to patients with IHD, thereby excluding PAUSE-SCD, and incorporated individual patient data (IPD) from the ERASE study, which was not formally published or available to us. The follow-up duration also varied, at 3 years for the present study vs. 4 years in the prior meta-analysis. However, neither our subgroup analysis of IHD studies nor the meta-regression by proportion of ischaemic patients demonstrated a significant effect on mortality in studies exclusively or predominantly with IHD patients, suggesting the observed difference cannot solely be explained by the exclusion of non-ischaemic patients. Potential study-specific factors in the three trials that also included patients with NICM^11,29,34^ that may explain our results are described in a few paragraphs below. A detailed comparison with previously published systematic reviews is presented in the Supplementary Material section (Supplementary material online, Table  *S*-6).

At the individual trial level, only PARTITA detected a reduction in mortality following CA.^29^ There were no deaths in the ablation group but a relatively high mortality in the control group (33%). The ablation group contained fewer patients with a background of diabetes (41% vs. 19%), kidney disease (27% vs. 14%) and chronic obstructive pulmonary disease (23% vs. 9.5%), which may explain the findings not replicated elsewhere.

Our meta-analysis demonstrates significant reductions in VT storm, cardiovascular hospitalizations, and ICD therapies, indicating a meaningful morbidity benefit. With increasing emphasis on patient-centred care and the improving safety profile of catheter ablation, the potential for fewer hospitalizations and ICD shocks represents an important clinical consideration that may substantially enhance patients’ quality of life, warranting intervention even in the absence of a proven mortality benefit. By incorporating a larger and more diverse dataset, including patients with NICM, findings of this meta-analysis extend and reinforce previous meta-analyses, further reinforcing the role of catheter ablation in the contemporary management of VT.

This review shows a reduction in CV hospitalization with CA, but with imprecision (a broad 95%CI), so the exact effect size is uncertain. The reduction is driven by positive results from PREVENTIVE-VT, SURVIVE VT and VTACH, with others reporting neutral results. Notably, there was a sizeable difference between the lowest and highest reported rates of CV hospitalization (4.3% in the PARTITA ablation group vs. 54.6% in VTACH controls). Heterogeneity was not explained by subgroup analysis of AAD use or ablation type, but sensitivity analysis revealed studies before 2020 had a lower heterogeneity than those from 2020–2024 (*I^2^* = 0% vs. *I^2^* = 63%). Later studies recruited patients with both IHD and NICM, as well as patients meeting both primary and secondary prevention ICD criteria, and their mixed comorbidity will be reflected in higher heterogeneity between studies.

There was a significant reduction in ICD therapy and ICD shocks, but with moderate heterogeneity for both (*I^2^* = 33% and *I^2^* = 44% respectively). Some studies (e.g. VANISH-2, SURVIVE-VT) were designed as a direct comparison of AADs and ablation, and as such no class I or III AADs were used in the ablation arm, whilst other RCTs, such as PAUSE-SCD, allowed baseline use of AADs in the ablation group with escalated doses in the control arm. PAUSE-SCD advised additional AAD ‘at the discretion of the treating physician and based on local practice, which is likely to vary significantly in a multicentre, international study. This variation reflects real-world practice and goes some way to explaining the heterogeneity between study results. The disparity in protocols also means question relating to CA being used as an alternative to, or in conjunction with AADs, go unanswered, as there is too much variation in timing, dosing and types of AADs used to assimilate this information. Pragmatically, given how high-risk these patients are for deterioration, AADs will continue to be used alongside CA in those who tolerate them.

While it is commonly accepted that VT ablation in patients with IHD has a lower recurrence rate than in NICM,^37^ our meta-regression demonstrated a higher proportion of IHD was significantly associated with a smaller relative benefit of ablation for VT recurrence (coefficient = 0.007, *P* = 0.04) (see Supplementary material online, Table  *S*-15). However, these results should be interpreted with caution due to additional study-specific factors in the three trials that included patients with NICM, which may have influenced the outcomes and could not be accounted for in the univariate meta-regression. These three trials were among those demonstrating a more pronounced benefit of VT ablation compared with controls for VT recurrence. PARTITA^29^ included approximately 19% of patients with NICM, and no AADs were used in the control group – consistent with our subgroup analysis showing a greater benefit of ablation in studies without AAD use. PAUSE-SCD^34^ included 31% of patients with NICM and 34% with ARVC; epicardial ablation was encouraged per protocol and performed in 55% of patients, which likely contributed to the observed benefit, as ablation of ARVC has been associated with better outcomes compared with other forms of NICM.^38^  *Epstein et al.*^11^ had the shortest follow-up period (six months), and shorter follow-up durations have been shown to inflate the apparent efficacy of VT ablation.^39^ Longer follow-up, as observed in most trials including only IHD patients (e.g. VANISH-2 had a median of 52 months), allows progression or development of new substrate leading to recurrent VT. Importantly, VT recurrence was not measured uniformly (see Supplementary material online, Table  *S*-18), which can also explain observed differences for this endpoint across the different trials. No significant associations were observed in the meta-regression assessing IHD as a study-level moderator for the other outcomes.

There was variation in ICD programming between studies (see Supplementary material online, Table  *S*-18). More aggressive programming leads to more therapies, not all of which will be necessary. VANISH, which advised a VT detection zone of 150 beats per minute (bpm) reported a high shock rate (42.5% both groups), but SURVIVE VT with a recommended VT detection zone of 185bpm reported lower rates (25.4 and 21.9%). The MADIT-RIT trial (2012) demonstrated improved all-cause mortality and a reduction in inappropriate therapies with higher rate or delayed detection zones compared with conventional programming.^33^ Studies, where recruitment preceded MADIT-RIT, such as VANISH and SMS, encouraged lower detection zones, meaning some therapies would not have occurred had higher thresholds been used. Indeed, this is reflected in real-world data. Ruwald et al reported a significant reduction in appropriate therapies between 2007 and 2016 from 28.2 to 7.9 therapies per 100 person years (*P* < 0.001), a reflection of both improved heart failure therapies and ICD programming.^40^

The significant reduction in the primary endpoint in SURVIVE-VT (composite of CV death, heart failure hospitalization, appropriate ICD shock and significant treatment complications) was driven by a reduction in treatment-related complications (9.9% vs. 28.8%, *P* = 0.006), the majority of which were AAD side effects. The majority of CA studies focus on procedural safety rather than drug side effects (see Supplementary material online, Table  *S*-19). It is difficult to compare safety of each intervention directly when the treatments are so different. Procedure-related vascular injury or tamponade are easily measured whereas drug side effects such as pulmonary toxicity may happen years after initiation (even outside the study follow up period), so are likely underrepresented in most studies, which may bias any risk vs. benefit analysis.

As ongoing VT trials shift their focus towards newer therapies such stereotactic arrhythmia radioablation or autonomic modulation,^41^ this study consolidates a growing body of evidence confirming an essential role for CA in patients with structural heart disease, whilst newer techniques are yet to be validated through RCTs.^42^

### Limitations

Our systematic review followed high-rigour methodology, with strict adherence to PRISMA and Cochrane methodology, providing a detailed appraisal of evidence with GRADE methodology for the first time. However, some limitations that are inherent to the data need to be highlighted. Firstly, the lack of patient diversity and hence the generalizability of the data. The majority of patients were males (females account for <10%) with a background of IHD reflecting the persistent underrepresentation of women in cardiovascular research. This sex imbalance limits the generalisability of our findings, as sex-related differences in arrhythmia substrate, ablation response, and outcomes remain incompletely understood,^43^ and as such increased recruitment of women (or a study recruiting only women) is of the utmost important for the field moving forward. Secondly, heterogeneity was observed for AAD use, ICD programming protocols and VT ablation strategy. Where available, subgroup analyses were performed, but this was not possible in some instances (including for patients with NICM only or based AAD type). It is also recognised that combining trials with differing baseline exposures within subgroup definitions reduces interpretability. However, the large number of covariates relative to the limited number of included studies precluded the use of multivariable analysis. Therefore, several questions regarding optimal patient selection and procedural protocols remain unanswered.

A 2019 meta-analysis of 1138 patients, from RCTs as well as non-randomized studies, in which 44% of patients underwent an endo-epicardial approach, found there was significant benefit of endo-epicardial procedures compared with endocardial procedures alone. Interestingly, the effect was largest in patients with IHD, where there was a significant reduction in VT recurrence or appropriate ICD therapy (OR 0.39, 95%CI 0.18–0.83) and all-cause mortality (OR 0.38 95%CI 0.15–0.99).^44^ It is possible the full benefit of combined endo-epicardial procedures is underestimated in our meta-analysis due to lack of statistical power, as the vast majority of procedures were endocardial only. Thirdly, most studies focus on hard outcomes relating to mortality and device therapies so there is limited data on how ablation affects quality of life. SMS used the 36 item short form survey (SF-36),^45^ and found no difference in the scores relating to general health, physical health or mental health between groups. A VANISH sub-study also found no overall difference in health-related quality of life when using four validated questionnaires- the SF-36, the implanted cardioverter defibrillator (ICD) Concerns questionnaire (ICDC), the Hospital Anxiety and Depression Scale (HADS), and the EuroQol five dimensions questionnaire (EQ-5D).^46^

Finally, all studies to-date lack sham-procedure control groups. Even though lack of blinding may be less of an issue for truly objective outcomes like mortality or appropriate ICD shocks, unblinded trials may lead to differences in subsequent patient management, for example more aggressive AADs in patients who do not undergo ablation, exposing them to more adverse drug effects. However, due to slow enrolment in VT trials adding a sham procedure arm would add further complexity, and may not be a realistic prospect. If sham-controlled VT trials prove too challenging, studies in other fields – such as the recent SHAM-PVI trial^47^ in atrial fibrillation – may offer insights into the placebo effects of sham ablation procedures more broadly, although their generalisability to VT populations is uncertain.

## Conclusion

In this largest-to-date meta-analysis, our pooled estimates hint at a potential mortality reduction effect of catheter ablation, which requires further confirmation in a large and properly powered RCT. No reduction in cardiovascular mortality was found. A clear reduction in VT recurrence, VT storm, ICD therapies and CV hospitalizations was found in patients with structural heart disease treated with catheter ablation as opposed to standard therapy.

## Supplementary Material

oeaf171_Supplementary_Data

## Data Availability

No new data were generated or analysed in support of this research. All data were extracted from publications or provided by study authors and made available on the manuscript.

## Data sharing

Datasets generated from this review are available from the corresponding author upon request.
