# Correction to: Outcomes of catheter ablation for ventricular tachycardia in structural heart disease: a meta-analysis and quality appraisal of trials

**DOI:** 10.1093/ehjopen/oeag096

**Published:** 2026-06-04

This is a correction to: Debbie Falconer, Ahmed Salih, Gabriella Captur, Richard J Schilling, Pier D Lambiase, Nikos Papageorgiou, Rui Providencia, Outcomes of catheter ablation for ventricular tachycardia in structural heart disease: a meta-analysis and quality appraisal of trials, *European Heart Journal Open*, Volume 6, Issue 1, January 2026, oeaf171, https://doi.org/10.1093/ehjopen/oeaf171

The title has been corrected to read: “Outcomes of catheter ablation for ventricular tachycardia in structural heart disease: a meta-analysis and quality appraisal of trials”.

