# Supplementary material for: Outcomes of catheter ablation for ventricular tachycardia in structural heart disease: a meta-analysis and quality appraisal of trials
Source: Eur Heart J Open. 2025 Dec 11;6(1):oeaf171. doi: 10.1093/ehjopen/oeaf171 (PMC12798810; doi:10.1093/ehjopen/oeaf171)

**Table S-1**: Preferred Reporting Items for Systematic Reviews and Meta-Analysis checklist.

| **Section and Topic** | **Item #** | **Checklist item** | **Location where item is reported (page no).** |
| --- | --- | --- | --- |
| **TITLE** | | |  |
| Title | 1 | Identify the report as a systematic review. | Title- P1 |
| **ABSTRACT** | | |  |
| Abstract | 2 | See the PRISMA 2020 for Abstracts checklist. | Abstract- P2 |
| **INTRODUCTION** | | |  |
| Rationale | 3 | Describe the rationale for the review in the context of existing knowledge. | Introduction- P3-4 |
| Objectives | 4 | Provide an explicit statement of the objective(s) or question(s) the review addresses. | Introduction- P4 |
| **METHODS** | | |  |
| Eligibility criteria | 5 | Specify the inclusion and exclusion criteria for the review and how studies were grouped for the syntheses. | Methods- P4-5 |
| Information sources | 6 | Specify all databases, registers, websites, organisations, reference lists and other sources searched or consulted to identify studies. Specify the date when each source was last searched or consulted. | Methods- P4 |
| Search strategy | 7 | Present the full search strategies for all databases, registers and websites, including any filters and limits used. | Methods- P4 |
| Selection process | 8 | Specify the methods used to decide whether a study met the inclusion criteria of the review, including how many reviewers screened each record and each report retrieved, whether they worked independently, and if applicable, details of automation tools used in the process. | Methods- P5 |
| Data collection process | 9 | Specify the methods used to collect data from reports, including how many reviewers collected data from each report, whether they worked independently, any processes for obtaining or confirming data from study investigators, and if applicable, details of automation tools used in the process. | Methods- P5 |
| Data items | 10a | List and define all outcomes for which data were sought. Specify whether all results that were compatible with each outcome domain in each study were sought (e.g. for all measures, time points, analyses), and if not, the methods used to decide which results to collect. | Methods- P5 |
|  | 10b | List and define all other variables for which data were sought (e.g. participant and intervention characteristics, funding sources). Describe any assumptions made about any missing or unclear information. | Methods- P5 |
| Study risk of bias assessment | 11 | Specify the methods used to assess risk of bias in the included studies, including details of the tool(s) used, how many reviewers assessed each study and whether they worked independently, and if applicable, details of automation tools used in the process. | Methods- P5 |
| Effect measures | 12 | Specify for each outcome the effect measure(s) (e.g. risk ratio, mean difference) used in the synthesis or presentation of results. | Methods- P6 |
| Synthesis methods | 13a | Describe the processes used to decide which studies were eligible for each synthesis (e.g. tabulating the study intervention characteristics and comparing against the planned groups for each synthesis (item #5)). | Methods- P6-7 |
|  | 13b | Describe any methods required to prepare the data for presentation or synthesis, such as handling of missing summary statistics, or data conversions. | Methods- P6-7 |
|  | 13c | Describe any methods used to tabulate or visually display results of individual studies and syntheses. | Methods- P6-7 |
|  | 13d | Describe any methods used to synthesize results and provide a rationale for the choice(s). If meta-analysis was performed, describe the model(s), method(s) to identify the presence and extent of statistical heterogeneity, and software package(s) used. | Methods- P6-7 |
|  | 13e | Describe any methods used to explore possible causes of heterogeneity among study results (e.g. subgroup analysis, meta-regression). | Methods- P6-7 |
|  | 13f | Describe any sensitivity analyses conducted to assess robustness of the synthesized results. | Methods- P5-6 |
| Reporting bias assessment | 14 | Describe any methods used to assess risk of bias due to missing results in a synthesis (arising from reporting biases). | Methods P5 |
| Certainty assessment | 15 | Describe any methods used to assess certainty (or confidence) in the body of evidence for an outcome. | Methods P6 |
| **RESULTS** | | |  |
| Study selection | 16a | Describe the results of the search and selection process, from the number of records identified in the search to the number of studies included in the review, ideally using a flow diagram. | Figure 1- P22 |
|  | 16b | Cite studies that might appear to meet the inclusion criteria, but which were excluded, and explain why they were excluded. | Supp appendix: P3 |
| Study characteristics | 17 | Cite each included study and present its characteristics. | Table 1- Page 27 |
| Risk of bias in studies | 18 | Present assessments of risk of bias for each included study. | Results- P8-9 |
| Results of individual studies | 19 | For all outcomes, present, for each study: (a) summary statistics for each group (where appropriate) and (b) an effect estimate and its precision (e.g. confidence/credible interval), ideally using structured tables or plots. | Results- P7-10 |
| Results of syntheses | 20a | For each synthesis, briefly summarise the characteristics and risk of bias among contributing studies. | Results P8-9 and Supp appendix P6 |
|  | 20b | Present results of all statistical syntheses conducted. If meta-analysis was done, present for each the summary estimate and its precision (e.g. confidence/credible interval) and measures of statistical heterogeneity. If comparing groups, describe the direction of the effect. | Results P9-12 and Figure 2-4 |
|  | 20c | Present results of all investigations of possible causes of heterogeneity among study results. | Results P9-12 |
|  | 20d | Present results of all sensitivity analyses conducted to assess the robustness of the synthesized results. | Results P11-12 and Supp Appendix P9-16 |
| Reporting biases | 21 | Present assessments of risk of bias due to missing results (arising from reporting biases) for each synthesis assessed. | Supp Appendix P19 and 25 |
| Certainty of evidence | 22 | Present assessments of certainty (or confidence) in the body of evidence for each outcome assessed. | Results P11 |
| **DISCUSSION** | | |  |
| Discussion | 23a | Provide a general interpretation of the results in the context of other evidence. | Discussion P12-14 |
|  | 23b | Discuss any limitations of the evidence included in the review. | Discussion P15-16 |
|  | 23c | Discuss any limitations of the review processes used. | Discussion P15 |
|  | 23d | Discuss implications of the results for practice, policy, and future research. | Discussion P12 |
| **OTHER INFORMATION** | | |  |
| Registration and protocol | 24a | Provide registration information for the review, including register name and registration number, or state that the review was not registered. | Methods P4 |
|  | 24b | Indicate where the review protocol can be accessed, or state that a protocol was not prepared. | Methods P4 |
|  | 24c | Describe and explain any amendments to information provided at registration or in the protocol. | Methods P4 |
| Support | 25 | Describe sources of financial or non-financial support for the review, and the role of the funders or sponsors in the review. | Title P1 |
| Competing interests | 26 | Declare any competing interests of review authors. | Title P1 |
| Availability of data, code and other materials | 27 | Report which of the following are publicly available and where they can be found: template data collection forms; data extracted from included studies; data used for all analyses; analytic code; any other materials used in the review. | Data sharing statement after references |

**Table S-2:** Excluded studies.

| Study title | Published Year | Notes | Source |
| --- | --- | --- | --- |
| Catheter Ablation Versus Amiodarone for Shock Prophylaxis in Defibrillator Patients With Ventricular Tachycardia | 2010 | Trial terminated due "anticipated non-feasibility of recruitment objectives" | NCT01097330 |
| Early Ablation Therapy for the Treatment of Ischemic Ventricular Tachycardia in Patients With Implantable Cardioverter Defibrillators (ASPIRE) | 2012 | Terminated trial with no published results | NCT01557842 |
| Anti-arrhythmic Medication v. MRI-Merge Ablation in the Treatment of Ventricular Tachycardia | 2008 | Trial withdrawn – ‘principal investigator left the institution’ | NCT00721032 |
| Catheter ablation versus Amiodarone to prevent Future ventricular tachycardia Episodes in patients with a defibrillator and a history of a myocardial infarction (CARFE-2) | 2011 | Suspended trial | NL-OMON35926 |
| Trial Comparing Ablation With Medical Therapy in Patients With Ventricular Tachycardia (VeTAMed) | 2013 | Withdrawn trial due to low recruitment | NCT01798277 |

**Table S-3:**  Ongoing trials of catheter ablation of ventricular tachycardia

| Registration | Title (acronym) | Population | Comparison |
| --- | --- | --- | --- |
| ACTRN12620000045910 | Catheter ablation vs anti-arrhythmic drugs for ventricular tachycardia (CAAD-VT) | Structural heart disease with sustained or inducible VT undergoing catheter ablation | Catheter ablation vs AADs |
| NCT06556485 | Preventive Catheter Ablation for Ventricular arrhythmiaS in Patients With End-sTage Heart faiLure (CASTLE-VT) | Patients with advanced heart failure awaiting heart transplant. | Preventative ablation vs medical therapy |

Abbreviations: AAD, anti-arrhythmic drugs; ICD, implantable cardiac defibrillator; VT, ventricular tachycardia

**Table S-4.** Supplementary material- baseline characteristics.

| Study | Group | Time after MI, years  Mean±SD or Median (IQR) | Previous PCI  N (%) | Previous CABG  N (%) | Hypertension  N (%) | Diabetes mellitus  N (%) | CKD stage 3 or less N (%) | Previous stroke  N (%) |
| --- | --- | --- | --- | --- | --- | --- | --- | --- |
| Epstein 1998 | Ablation  Control |  |  |  |  |  |  |  |
| Reddy 2007 | Ablation  Control | 8.8±8.5  7.9±7.8 | All revasc 46 (72)  All revasc 40 (62) | | 47 (73)  43 (67) | 24 (38)  32 (50) |  | 3 (5)  8 (12) |
| Kuck 2010 | Ablation  Control | 12.6±8  13.3±8.6 | 26 (50)  24 (44) | 26 (50)  22 (40) |  |  |  |  |
| Al-Khatib 2014 | Ablation  Control |  | 6 (46)  8 (57) | 8 (62)  8 (57) | 9 (69)  12 (86) | 6 (46)  5 (36) |  |  |
| Saap 2016 | Ablation  Control | 15.7±9.4  15.7±9.8 | 50 (37.9)  62 (49) | 63 (47.7)  55 (43.3) | 92 (69.7)  88 (69.3) | 37 (28)  40 (31.5) |  |  |
| Kuck 2017 | Ablation  Control | 11.1±6.6  8.6±7.8 | 23 (46)  25 (46) | 21 (41)  24 (43) |  |  |  |  |
| Willems, 2020 | Ablation  Control | 10.3±12  9.2±9.1 | 39 (51.3)  47 (56.6) | 22 (28.9)  17 (20.5) | 62 (81.6)  66 (79.5) | 23 (30.3)  22 (26.5) | 14 (18.4)  13 (15.7) |  |
| Tung, 2022 | Ablation  Control |  |  |  | 19 (31.7)  21 (34.4) | 8 (13.3)  15 (24.6) | 6 (10)  6 (9.8) |  |
| Bella Della, 2022 | Ablation  Control |  |  |  | 17 (81)  15 (68) | 4 (19)  9 (41) | 3 (14)  6 (27) | 2 (9.5)  1 (4.5) |
| Arenal, 2022 | Ablation  Control | 14 (6–24)  14 (7–23) | 26 (38.2)  26 (37.1) | 18 (26.5)  12 (17.1) | 56 (78.9)  47 (64.4) | 21 (29.6)  15 (20.5) | 8 (11.3)  7 (9.6) |  |
| Žižek, 2024 | Ablation  Control | 65 (57–73)  71 (66–76) | 13(43.3)  13(43.3) | 1(3.3)  4 (13.3) | 24 (80)  25 (83.3) | 8 (26.7)  9 (30) | 6(20)  6(20) |  |
| Sapp, 2024 | Ablation  Control | 13.3±9.9  14.8±10.4 | 128 (63.1)  121 (56.8) | 82 (40.4)  88 (41.3) | 160 (78.8)  169 (79.3) | 79 (38.9)  83 (39) | 31 (15.3)  23 (10.8) |  |

Abbreviations: CABG: coronary artery bypass graft; CKD: chromic kidney disease;; MI: myocardial infarction; PCI: percutaneous coronary intervention.

**Table S-5:** Summary of findings

| Outcome | Number Of Studies  (& Patients) | Event Rate | NNT to Prevent one Event | Risk Ratio (95% CI) | p-value | Indirectness  Imprecision  Publication Bias | Heterogeneity & Risk of Bias | GRADE |
| --- | --- | --- | --- | --- | --- | --- | --- | --- |
| All-cause Mortality | 12 (1630) | 15.7% vs 18.4% | Non-significant | 0.87 (0.70 to 1.08) | 0.20 | No Indirectness*  Imprecision  ↓1 level  No Publication Bias | Low heterogeneity (I^2^= 0%)  No RoB** | Moderate Certainty  ⊕⊕⊕O |
| Cardiovascular Mortality | 9  (1446) | 9.5 % vs 10.8% | Non-significant | 0.89  (0.65 to 1.21) | 0.46 | No Indirectness*  Imprecision  ↓1 level  N.A. (<10 RCT) | Low heterogeneity (I^2^=0%)  RoB – ↓1 level  (Performance) | Low Certainty  ⊕⊕OO |
| VT Recurrence | 10  (1285) | 45.7% vs 53.1% | 13.6 patients | 0.83  (0.72 to 0.95) | 0.01 | No Indirectness*  Imprecision  ↓1 level  No Publication Bias | Low heterogeneity (I^2^= 21%)  No RoB** | ⊕⊕⊕O |
| VT Storm | 8  (1272) | 17.5% vs 22.7% | 17.9 patients | 0.78  (0.63 to 0.97) | 0.03 | No Indirectness*  Imprecision  ↓1 level  N.A. (<10 RCT) | Low heterogeneity (I^2^=0%)  No RoB** | Moderate Certainty  ⊕⊕⊕O |
| Cardiovascular Hospitalization | 10  (1451) | 33.5% vs 41.8% | 12.0 patients | 0.78  (0.65 to 0.94) | 0.01 | No Indirectness*  Imprecision  ↓1 level  No Publication Bias | Moderate heterogeneity (I^2^=41%)  RoB – ↓1 level  (Performance | Low Certainty  ⊕⊕OO |
| Appropriate ICD Therapy | 6  (706) | 29.7% vs. 41.4% | 8.5 patients | 0.74  (0.61 to 0.89) | 0.002 | No Indirectness*  Imprecision  ↓1 level  N.A. (<10 RCT) | Moderate heterogeneity (I^2^=33%)  No RoB** | Moderate Certainty  ⊕⊕OO |
| Appropriate ICD Shocks | 10  (1549) | 23.9% vs. 33.2% | 10.8 patients | 0.67  (0.52-0.86) | 0.002 | No Indirectness*  Imprecision  ↓1 level  N.A. (<10 RCT) | Moderate heterogeneity (I^2^=44%)  No RoB** | Moderate Certainty  ⊕⊕OO |
| Appropriate ATP | 6  (1143) | 40.9% vs. 45.3% | 16.3 patients | 0.88  (0.74-1.06) | 0.18 | No Indirectness*  Imprecision  ↓1 level  N.A. (<10 RCT) | Moderate heterogeneity (I^2^=26%)  No RoB** | Moderate Certainty  ⊕⊕⊕O |

NNT: Number Needed to Treat. Other abbreviations as per Table S-1. Note: * downgrade by one level may be considered if applying the evidence to non-ischemic cardiomyopathy or women, as these were under-represented in the included RCTs; ** No downgrade as outcome was considered objective/no due to subjective interpretation and unlikely to be affected by lack of blinding. **

**Table S-6:** Reconstructed all-cause mortality survival analysis at 12, 24 and 36 months

| Follow-up (year) | Hazard Ratio (95% CI) | p-value | Heterogeneity  p-value |
| --- | --- | --- | --- |
| 1 | 0.74 (0.48 to 1.16) | 0.19 | 0.0019 |
| 2 | 0.80 (0.55 to 1.16) | 0.23 | < 0.001 |
| 3 | 0.79 (0.57 to 1.11) | 0.17 | 0.003 |

**Table S-7**. Subgroup analysis with only studies of patients with ischemic heart disease

| Outcome | Number Of Studies | Number of Patients | Number of Events | Risk Ratio (95% CI) | p-value | Heterogeneity analysis (*I^2^*) |
| --- | --- | --- | --- | --- | --- | --- |
| VT Recurrence | 7 | 1012 | 505 | 0.91 (0.81 to 1.03) | 0.14 | 0 |
| VT Storm | 7 | 1225 | 248 | 0.78 (0.63 to 0.98) | 0.03 | 0 |
| All-cause Mortality | 10 | 1462 | 261 | 0.88 (0.71 to 1.09) | 0.22 | 0 |
| Cardiovascular Hospitalization | 8 | 1283 | 505 | 0.77 (0.62 to 0.95) | 0.02 | 0.50 |
| Cardiovascular Mortality | 7 | 1277 | 139 | 0.92 (0.67 to 1.26) | 0.60 | 0 |
| Appropriate ICD Therapy | 6 | 706 | 252 | 0.74 (0.61 to 0.89) | 0.002 | 0.32 |

Abbreviations as per previous tables.

**Table S-8**. Subgroup analysis with only studies of ablation as a secondary prevention strategy

| Outcome | Number Of Studies | Number of Patients | Number of Events | Risk Ratio (95% CI) | p-value | Heterogeneity analysis (*I^2^*) |
| --- | --- | --- | --- | --- | --- | --- |
| VT Recurrence | 9 | 1164 | 584 | 0.85 (0.74 to 0.98) | 0.02 | 0.15 |
| VT Storm | 9 | 1164 | 584 | 0.85 (0.74 to 0.98) | 0.02 | 0 |
| All-cause Mortality | 10 | 1449 | 249 | 0.88 (0.70 to 1.10) | 0.27 | 0 |
| Cardiovascular Hospitalization | 8 | 1270 | 490 | 0.82 (0.68 to 0.98) | 0.03 | 0.23 |
| Cardiovascular Mortality | 7 | 1264 | 130 | 0.95 (0.69 to 1.31) | 0.76 | 0 |
| Appropriate ICD Therapy | 5 | 646 | 235 | 0.76 (0.62 to 0.92) | 0.006 | 0.32 |

Abbreviations as per previous tables.

**Table S-9** Subgroup analysis with only published trials excluding Epstein et al and ERASE-VT

| Outcome | Number Of Studies | Number of Patients | Number of Events | Risk Ratio (95% CI) | p-value | Heterogeneity analysis (*I^2^*) |
| --- | --- | --- | --- | --- | --- | --- |
| VT Recurrence | 11 | 1579 | 272 | 0.88 (0.71 to 1.09) | 0.24 | 0 |
| VT Storm | 9 | 1446 | 147 | 0.89 (0.65 to 1.21) | 0.46 | 0 |
| All-cause Mortality | 8 | 1129 | 550 | 0.88 (0.77 to 1.00) | 0.06 | 0.09 |
| Cardiovascular Hospitalization | 8 | 1272 | 250 | 0.78 (0.63 to 0.97) | 0.03 | 0.05 |
| Cardiovascular Mortality | 10 | 1451 | 547 | 0.78 (0.65 to 0.94) | 0.01 | 0.41 |
| Appropriate ICD Therapy | 6 | 706 | 252 | 0.74 (0.61 to 0.89) | 0.002 | 0.32 |

Abbreviations as per previous tables.

**Table S-10**. Subgroup analysis based on the follow-up period

| Outcome | Subgroup | Number of Studies | Number of Patients | Number of Events | Risk Ratio (95% CI) | p-value for subgroup Difference |
| --- | --- | --- | --- | --- | --- | --- |
| VT Recurrence | <=6 months | 2 | 132 | 74 | 0.90 (0.43 to 1.91) | 0.84 |
|  | 12-24 months | 2 | 263 | 136 | 0.79 (0.62 to 0.99) |  |
|  | >24 months | 4 | 695 | 360 | 0.86 (0.68 to 1.09) |  |
| VT Storm | <=6 months | 0 | NA | NA | NA (NA to NA) | 0.44 |
|  | 12-24 months | 2 | 235 | 46 | 0.58 (0.25 to 1.35) |  |
|  | >24 months | 5 | 893 | 197 | 0.82 (0.64 to 1.05) |  |
| All-cause Mortality | <=6 months | 1 | 27 | 4 | 1.08 (0.18 to 6.57) | 0.96 |
|  | 12-24 months | 3 | 394 | 34 | 0.98 (0.38 to 2.54) |  |
|  | >24 months | 6 | 1014 | 227 | 0.88 (0.70 to 1.11) |  |
| Cardiovascular Hospitalization | <=6 months | 1 | 27 | 12 | 0.77 (0.32 to 1.83) | 0.91 |
|  | 12-24 months | 2 | 266 | 98 | 0.79 (0.46 to 1.38) |  |
|  | >24 months | 6 | 1014 | 397 | 0.88 (0.75 to 1.02) |  |
| Cardiovascular Mortality | <=6 months | 0 | NA | NA | NA (NA to NA) | 0.23 |
|  | 12-24 months | 2 | 287 | 13 | 0.45 (0.14 to 1.43) |  |
|  | >24 months | 6 | 1015 | 128 | 0.94 (0.68 to 1.30) |  |
| Appropriate ICD Therapy | <=6 months | 0 | NA | NA | NA (NA to NA) | 0.98 |
|  | 12-24 months | 3 | 391 | 157 | 0.68 (0.54 to 0.86) |  |
|  | >24 months | 2 | 171 | 61 | 0.67 (0.33 to 1.36) |  |

Abbreviations as per previous tables.

**Table S-11**. Subgroup analysis based on AAD use

| Outcome | Subgroup | Number of Studies | Number of Patients | Number of Events | Risk Ratio (95% CI) | p-value for subgroup Difference |
| --- | --- | --- | --- | --- | --- | --- |
| VT Recurrence | No AAD use | 1 | 47 | 19 | 0.61 (0.29 to 1.27) | 0.56 |
|  | Control arm only/escalating dose in control | 4 | 708 | 344 | 0.91 (0.73 to 1.14) |  |
|  | AAD in both treatment arms | 3 | 374 | 187 | 0.84 (0.68 to 1.02) |  |
| VT Storm | No AAD use | 3 | 235 | 24 | 0.27 (0.10 to 0.70) | 0.08 |
|  | Control arm only/escalating dose in control | 3 | 819 | 185 | 0.84 (0.66 to 1.08) |  |
|  | AAD in both treatment arms | 2 | 218 | 41 | 0.76 (0.44 to 1.31) |  |
| All-cause Mortality | No AAD use | 3 | 235 | 45 | 0.56 (0.32 to 0.99) | 0.23 |
|  | Control arm only/escalating dose in control | 5 | 967 | 190 | 0.93 (0.73 to 1.20) |  |
|  | AAD in both treatment arms | 3 | 377 | 37 | 1.06 (0.57 to 1.97) |  |
| Cardiovascular Hospitalization | No AAD use | 2 | 107 | 25 | 0.25 (0.10 to 0.61) | 0.03 |
|  | Control arm only/escalating dose in control | 5 | 967 | 378 | 0.83 (0.68 to 1.03) |  |
|  | AAD in both treatment arms | 3 | 377 | 144 | 0.83 (0.60 to 1.14) |  |
| Cardiovascular Mortality | No AAD use | 3 | 235 | 25 | 0.43 (0.19 to 0.96) | 0.15 |
|  | Control arm only/escalating dose in control | 4 | 941 | 115 | 1.02 (0.73 to 1.43) |  |
|  | AAD in both treatment arms | 2 | 270 | 7 | 0.81 (0.18 to 3.63) |  |
| Appropriate ICD Therapy | No AAD use | 2 | 188 | 46 | 0.39 (0.22 to 0.70) | 0.03 |
|  | Control arm only/escalating dose in control | 1 | 144 | 34 | 1.16 (0.64 to 2.08) |  |
|  | AAD in both treatment arms | 3 | 374 | 172 | 0.76 (0.61 to 0.94) |  |

Abbreviations as per previous tables. Sub-groups: (i) No AADs: no class I or III used in either arm at baseline or as part of study treatment - SMASH-VT, PARTITA, & PREVENTIVE-VT; (ii) Control arm only: class I or III AADs were only used in the control arm only, or, if used also in the ablation arm at baseline, dose escalation was only allowed in the control group – VANISH, VANISH 2, SURVIVE-VT, ERASE-VT, PAUSE-SCD, & CALYPSO; (iii) Both arms at baseline: class I or III AADs were used in both treatment arms at baseline, and both arms were allowed to escalate - SMS, VTACH, & BERLIN-VT.

**Table S-12**. Subgroup analysis based on ablation strategy

| Outcome | Subgroup | Number of Studies | Number of Patients | Number of Events | Risk Ratio (95% CI) | p-value for subgroup Difference |
| --- | --- | --- | --- | --- | --- | --- |
| VT Recurrence | Endocardial only | 4 | 409 | 177 | 0.83 (0.68 to 1.03) | 0.71 |
|  | Endocardial and epicardial | 3 | 564 | 304 | 0.90 (0.63 to 1.29) |  |
| VT Storm | Endocardial only | 7 | 856 | 156 | 0.70 (0.53 to 0.93) | 0.23 |
|  | Endocardial and epicardial | 1 | 416 | 94 | 0.92 (0.65 to 1.32) |  |
| All-cause Mortality | Endocardial only | 7 | 856 | 152 | 0.83 (0.62 to 1.10) | 0.48 |
|  | Endocardial and epicardial | 3 | 564 | 112 | 0.90 (0.65 to 1.25) |  |
| Cardiovascular Hospitalization | Endocardial only | 6 | 728 | 230 | 0.63 (0.47 to 0.85) | 0.028 |
|  | Endocardial and epicardial | 3 | 564 | 266 | 0.93 (0.79 to 1.11) |  |
| Cardiovascular Mortality | Endocardial only | 6 | 749 | 85 | 0.75 (0.51 to 1.13) | 0.17 |
|  | Endocardial and epicardial | 2 | 538 | 59 | 1.16 (0.72 to 1.88) |  |
| Appropriate ICD Therapy | Endocardial only | 5 | 550 | 188 | 0.72 (0.52 to 0.99) | NA |
|  | Endocardial and epicardial | 0 | NA | NA | NA (NA to NA) |  |

Abbreviations as per previous tables.

**Table S-13**. Subgroup analysis based on risk of bias assessment

| Outcome | Subgroup | Number of Studies | Number of Patients | Number of Events | Risk Ratio (95% CI) | p-value for subgroup Difference |
| --- | --- | --- | --- | --- | --- | --- |
| VT Recurrence | Lower quality studies | 4 | 290 | 165 | 0.74 (0.61 to 0.90) | 0.13 |
|  | Higher quality studies | 6 | 995 | 469 | 0.89 (0.77 to 1.03) |  |
| VT Storm | Lower quality studies | 2 | 235 | 46 | 0.58 (0.25 to 1.35) | 0.46 |
|  | Higher quality studies | 6 | 1037 | 204 | 0.81 (0.63 to 1.03) |  |
| All-cause Mortality | Lower quality studies | 4 | 313 | 36 | 0.65 (0.34 to 1.23) | 0.34 |
|  | Higher quality studies | 8 | 1317 | 242 | 0.90 (0.72 to 1.13) |  |
| Cardiovascular Hospitalization | Lower quality studies | 2 | 134 | 59 | 0.63 (0.42 to 0.95) | 0.26 |
|  | Higher quality studies | 8 | 1317 | 488 | 0.82 (0.68 to 0.99) |  |
| Cardiovascular Mortality | Lower quality studies | 1 | 128 | 10 | 0.43 (0.12 to 1.58) | 0.26 |
|  | Higher quality studies | 8 | 1318 | 137 | 0.93 (0.68 to 1.28) |  |
| Appropriate ICD Therapy | Lower quality studies | 2 | 235 | 93 | 0.57 (0.31 to 1.05) | 0.31 |
|  | Higher quality studies | 4 | 471 | 159 | 0.81 (0.62 to 1.04) |  |

Abbreviations as per previous tables.

**Table S-14**. Subgroup analysis based on study year

| Outcome | Subgroup | Number of Studies | Number of Patients | Number of Events | Risk Ratio (95% CI) | p-value for subgroup Difference |
| --- | --- | --- | --- | --- | --- | --- |
| VT Recurrence | Up to 2019 | 4 | 350 | 192 | 0.83 (0.64 to 1.06) | 0.86 |
|  | 2020-2024 | 5 | 884 | 418 | 0.85 (0.70 to 1.03) |  |
| VT Storm | Up to 2019 | 4 | 605 | 141 | 0.74 (0.55 to 0.98) | 0.49 |
|  | 2020-2024 | 4 | 667 | 109 | 0.51 (0.19 to 1.39) |  |
| All-cause Mortality | Up to 2019 | 5 | 632 | 121 | 0.90 (0.65 to 1.23) | 0.86 |
|  | 2020-2024 | 6 | 947 | 151 | 0.86 (0.65 to 1.16) |  |
| Cardiovascular Hospitalization | Up to 2019 | 4 | 504 | 177 | 0.76 (0.60 to 0.97) | 0.73 |
|  | 2020-2024 | 6 | 947 | 370 | 0.70 (0.47 to 1.05) |  |
| Cardiovascular Mortality | Up to 2019 | 3 | 498 | 64 | 0.82 (0.52 to 1.29) | 0.92 |
|  | 2020-2024 | 6 | 948 | 83 | 0.85 (0.49 to 1.47) |  |
| Appropriate ICD Therapy | Up to 2019 | 3 | 346 | 137 | 0.70 (0.53 to 0.94) | 0.74 |
|  | 2020-2024 | 3 | 360 | 115 | 0.77 (0.50 to 1.19) |  |

Abbreviations as per previous tables.

**Table S-15**. Meta-regression result by % of ischemic cardiomyopathy participants

| Outcome | Covariate  co-efficient | Lower CI | Upper CI | p-value |
| --- | --- | --- | --- | --- |
| VT Recurrence | 0.007 | 0.0004 | 0.014 | 0.04 |
| VT Storm | 0.068 | -0.091 | 0.226 | 0.40 |
| All-cause Mortality | -0.003 | -0.023 | 0.016 | 0.73 |
| Cardiovascular Hospitalization | -0.002 | -0.014 | 0.011 | 0.77 |
| Cardiovascular Mortality | 0.007 | -0.012 | 0.034 | 0.58 |
| Appropriate ICD Therapy | - | - | - | - |

Abbreviations as per previous tables.

‘-‘ indicates an insufficient number of data points for meta-regression

**Table S-16**. Meta-regression result by proportion of male participants

| Outcome | Covariate  co-efficient | Lower CI | Upper CI | p-value |
| --- | --- | --- | --- | --- |
| VT Recurrence | 2.479 | −0.116 | 5.073 | 0.06 |
| Electrical Storm | 9.122 | −0.867 | 19.111 | 0.07 |
| All-cause Mortality | 0.260 | −6.058 | 6.578 | 0.94 |
| Cardiovascular Hospitalization | −1.489 | −6.966 | 3.988 | 0.59 |
| Cardiovascular Mortality | 6.761 | −3.409 | 16.931 | 0.19 |
| Appropriate ICD Therapy | −0.978 | −11.531 | 9.575 | 0.86 |

Abbreviations as per previous tables.

‘-‘ indicates an insufficient number of data points for meta-regression

**Table S-17.** Meta-regression by age

| Outcome | Covariate  co-efficient | Lower CI | Upper CI | p-value |
| --- | --- | --- | --- | --- |
| VT Recurrence | 0.029 | −0.011 | 0.069 | 0.15 |
| Electrical Storm | −0.091 | −0.482 | 0.300 | 0.65 |
| All-cause Mortality | −0.028 | −0.174 | 0.119 | 0.71 |
| Cardiovascular Hospitalization | −0.024 | −0.074 | 0.027 | 0.36 |
| Cardiovascular Mortality | 0.034 | −0.118 | 0.185 | 0.66 |
| Appropriate ICD Therapy | 0.118 | −0.050 | 0.286 | 0.17 |

Abbreviations as per previous tables.

‘-‘ indicates an insufficient number of data points for meta-regression

**Table S-18.**  ICD programming recommendations and definition of VT recurrence

| Study | VT1 zone | VT1 treatment 1/2 | VT2 zone | | VT2 treatment 1/2 | Further information provided in text | VT recurrence definition |
| --- | --- | --- | --- | --- | --- | --- | --- |
| Epstein 1998 |  |  | |  |  |  |  |
| Reddy 2007 |  |  | |  |  | ‘ICD programming standardised to ensure at least 1 VT zone in which ATP could occur’ | Sustained VT/VF or ICD-treated arrhythmia |
| Kuck 2010 | 60ms above slowest VT | ATP/ shock | |  |  |  | VT or VF |
| Al-Khatib 2014 | 20 beats lower than zone 2 | Monitor only | | 188bpm or 10 below slowest VT |  |  | Sustained VT and device therapies |
| Saap 2016 | 150bpm | ATP/ shock | |  | ATP/shock | *protocol changed to include prolonged detection halfway through | Documented VT events / ICD therapies |
| Kuck 2017 | 153-155 bpm  45 counts |  | | 172-180 bpm  31 counts |  | *detection counter lower in earlier protocols | VT or VF |
| Willems, 2020 | 153 bpm  45 counts |  | | 172 bpm  31 counts |  |  | VT, VF, or appropriate ICD therapy |
| Tung, 2022 | 185 or 10bpm slower than recorded  30 beats | APTx3/ shock | |  |  |  | Documented sustained VT or appropriate ICD therapy |
| NCT01182389 |  |  | |  |  |  | Any appropriate ICD therapy |
| Della Bella, 2022 | 130 bpm  32 beats | Monitor only | | >167 bpm  30 beats | ATPx2/ shock |  | ICD recorded arrhythmic events or ICD therapies |
| Arenal, 2022 | 15-20 lower than doc TV, max 188 bpm | ATPx4/ shock | | >188 bpm | ATPx1/ shock | *after MADIT RIT- delayed therapies strongly recommended | appropriate ICD therapy (shock or ATP) or documented sustained monomorphic VT >30 s |
| Žižek, 2024 | Monitor only |  | | Brand dependent: Approx. >185 bpm  30 intervals | ATPx1/ shock |  | sustained VT/VF or appropriate ICD therapy |
| Sapp, 2024 | Known VT:  >200 bpm- monitor only  <200 bpm- 20 below recorded  30 beats/ 7s | ATPx3/ 25J shock | | Brand dependent :  Approx. 181-240 bpm  30 beats/ 7 s | 1x ATP/ 30J shock |  | sustained VT or ICD therapy |

Abbreviation: ATP, anti-tachycardia pacing. BPM, beats per minute. Other abbreviations as per previous tables.

**Table S-19.** Complications across trials, inclusive of both ablation and device implantation procedures

| Study | Group | Major bleeding  N (%) | Stroke  N (%) | Vascular injury  N (%) | Tamponade/  perforation/ effusion  N (%) | Serious treatment-related events from AADs | Details of procedural complications (ablations and device implantations) |
| --- | --- | --- | --- | --- | --- | --- | --- |
| Epstein 1998 | Ablation  Control | 0  0 | 1 (1.4)  0 | 0  0 | 1 (1.4)  0 |  | Ablation group: 1x stroke, 1x perforation, 3x CHB |
| Reddy 2007 | Ablation  Control | 0  0 | 0  0 | 0  0 | 1 (1.5)  0 |  | Ablation group: 1x pericardial effusion, 1x CCF exacerbation, 1x DVT  No device-related complications. |
| Kuck 2010 | Ablation  Control | 0  0 | 0  0 | 0  0 | 0  0 |  | Ablation group: 2 procedures terminated early (transient ST elevation, TIA); 4 device complications  Control group: 9 device complications |
| Al-Khatib 2014 | Ablation  Control | 0  0 | 0  0 | 1  0 | 0  0 |  | Ablation group: 1x haematoma and 1x AKI |
| Saap 2016 | Ablation  Control | 3 (2.3)  1 (0.8) | 0  0 | 3 (2.8)  0 | 2 (1.5)  1 (0.8) | 4 (3)  6 (4.7) | Ablation group:: 3x bleeding, 3x vascular, injury, 2x cardiac perforation, 1x CHB  Control group: 1x bleed, 1x cardiac perforation |
| Kuck 2017 | Ablation  Control | 0  0 | 0  0 | 0  0 | 2 (3.7)  0 |  | Ablation group: 2x CHB, 2x effusion, 3x lead displacement  Control group: 1x pneumothorax, 2x lead displacement |
| Willems, 2020 | Ablation  Control | 3  0 | 0  0 | 3  1 | 3  0 |  | Ablation group: 1x perforation, 1x tamponade, 1x effusion, 2x groin bleed, 1x epistaxis, 1xCHB  Control group: 1x CHB, 1x thrombophlebitis |
| Tuck, 2022 | Ablation  Control | 0  0 | 0  0 | 1  0 | 3  0 |  | Ablation group: 1x Type B dissection, 1x aortic leaflet prolapse, 1x RV puncture, 1x LV perforation with drain, 1x late effusion  Control group: none |
| Della Bella, 2022 | Ablation  Control | 0  0 | 0  0 | 0  0 | 0  0 |  | No reported procedure complications |
| Arenal, 2022 | Ablation  Control | 0  0 | 2  0 | 1  0 | 1  0 | 0 (0)  21 (28.8) | Ablation group: 2x stroke; 1 effusion with drain; 1x cardiogenic shock; 1 pseudoaneurysm, 1x pulmonary oedema  Control group: none |
| Žižek, 2024 | Ablation  Control | 0  0 | 1  0 | 0  0 | 0  0 | 0  0 | Ablation group: 1xCHB requiring; 1x stroke with no residual neurological deficit.  No device-related complications. |
| Sapp, 2024 | Ablation  Control | 2  0 | 2  0 | 5  0 | 1  0 | 0  33 (15.4) | Ablation group: 2x deaths; 2x non-fatal stroke; 1x cardiac perforation; 5x vascular injury; 2x major bleeding  Control group: none |

Abbreviations: AKI: acute kidney injury; CCF: congestive cardiac failure; CHB: complete heart block; DVT: deep vein thrombosis; TIA: transient ischaemic attack. Other abbreviations as per Table 1.

**Table S-20.** Comparison of recent systematic reviews on catheter ablation of ventricular tachycardia and impact on outcomes

| Author, year | N | Cohort | Outcomes reported | PROSPERO Protocol | Subgroup/ sensitivity analysis performed | Use of individual patient data reconstruction? | Risk of bias performed? | GRADE published? | Summary of Findings | Meta-regression | PRISMA Checklist | List of Excluded Studies |
| --- | --- | --- | --- | --- | --- | --- | --- | --- | --- | --- | --- | --- |
| Falconer, 2025 | 1735 | IHD & non-IHD | 1) All-cause mortality  2) Cardiovascular mortality  3) VT recurrence  4) VT storm  5) Cardiac hospitalisations  6) ICD therapy  7) ICD shocks | CRD42024619649 | 1) IHD trials only  2) Secondary prevention studies only  3) Published trials only  4) Follow-up period  5) Drug use strategy  6) Ablation strategy  7) Risk of bias score  8) Study year | Yes | Yes | Yes | Yes | For % of ischaemic & % of ♂ participants | Yes | Yes |
| Reddy, 2025 | 1290 | IHD only | 1) All-cause mortality  2) VT recurrence  3) ICD shocks  4) Hospitalisation | CRD42024615654 | None reported | Yes | Yes | No | No | No | No | No |
| Reddy, 2023 | 874 | IHD only | 1) All-cause mortality  2) VT recurrence  3) ICD shocks  4) Hospitalisation | CRD42023390799 | 1) Ablation approach  2) % patients with prior MI | Yes | Yes | No | No | No | No | No |
| Ravi, 2022 | 2126 (non-randomised trials also included) | IHD & non-IHD | 1) All-cause mortality  2) VT recurrence  3) ICD shocks  4) Cardiac hospitalisations | No | 1) LVEF  2) Timing of ablation  3) Type of cardiomyopathy | No | Yes | No | No | No | No | No |
| Shalganov, 2022 | 1076 | IHD & non-IHD | 1) ICD therapy  2) ICD shocks  3) All-cause mortality  4) Cardiac hospitalisations  5) Complications | No | 1) LVEF | No | Yes | Yes | Yes | No | Yes | No |
| Lima da Silva, 2020 | 719 | IHD only | 1) All-cause mortality  2) Cardiovascular mortality  3) Recurrent VT/VF  4) VT storm  5) ICD therapies  6) ICD shocks | No | 1) Drug use strategy | No | Yes | Yes | Yes | No | No | No |

Reddy RK, Samways JW, Howard JP, Ahmad Y, Shun-Shin MJ, Saleh K, Naraen A, Kanagaratnam P, Whinnett ZI, Arnold AD. Catheter Ablation for Ventricular Tachycardia After Myocardial Infarction: An Updated Meta-Analysis of Randomized Controlled Trials. JACC Clin Electrophysiol. 2025;11:1047-1050. doi: 10.1016/j.jacep.2025.02.039.

Reddy RK, Howard JP, Ahmad Y, Shun-Shin MJ, Simader FA, Miyazawa AA, Saleh K, Naraen A, Samways JW, Katritsis G, Mohal JS, Kaza N, Porter B, Keene D, Linton NW, Francis DP, Whinnett ZI, Luther V, Kanagaratnam P, Arnold AD. Catheter Ablation for Ventricular Tachycardia After MI: A Reconstructed Individual Patient Data Meta-analysis of Randomised Controlled Trials. Arrhythm Electrophysiol Rev. 2023;12:e26. doi: 10.15420/aer.2023.07.

Ravi V, Poudyal A, Khanal S, Khalil C, Vij A, Sanders D, Larsen T, Trohman RG, Aksu T, Tung R, Santangeli P, Winterfield J, Sharma PS, Huang HD. A systematic review and meta-analysis comparing radiofrequency catheter ablation with medical therapy for ventricular tachycardia in patients with ischemic and non-ischemic cardiomyopathies. J Interv Card Electrophysiol. 2023;66:161-175. doi: 10.1007/s10840-022-01287-w.

Shalganov T, Stoyanov M, Traykov V. Outcomes of early catheter ablation for ventricular tachycardia in adult patients with structural heart disease and implantable cardioverter-defibrillator: An updated systematic review and meta-analysis of randomized trials. Front Cardiovasc Med. 2022;9:1063147. doi: 10.3389/fcvm.2022.1063147.

Lima da Silva G, Nunes-Ferreira A, Cortez-Dias N, de Sousa J, J Pinto F, Caldeira D. Radiofrequency catheter ablation of ventricular tachycardia in ischemic heart disease in light of current practice: a systematic review and meta-analysis of randomized controlled trials. J Interv Card Electrophysiol. 2020;59:603-616. doi: 10.1007/s10840-020-00870-3.

**Figure S-1**. Risk of bias assessment using the Cochrane ‘Risk of bias’ tool version 2


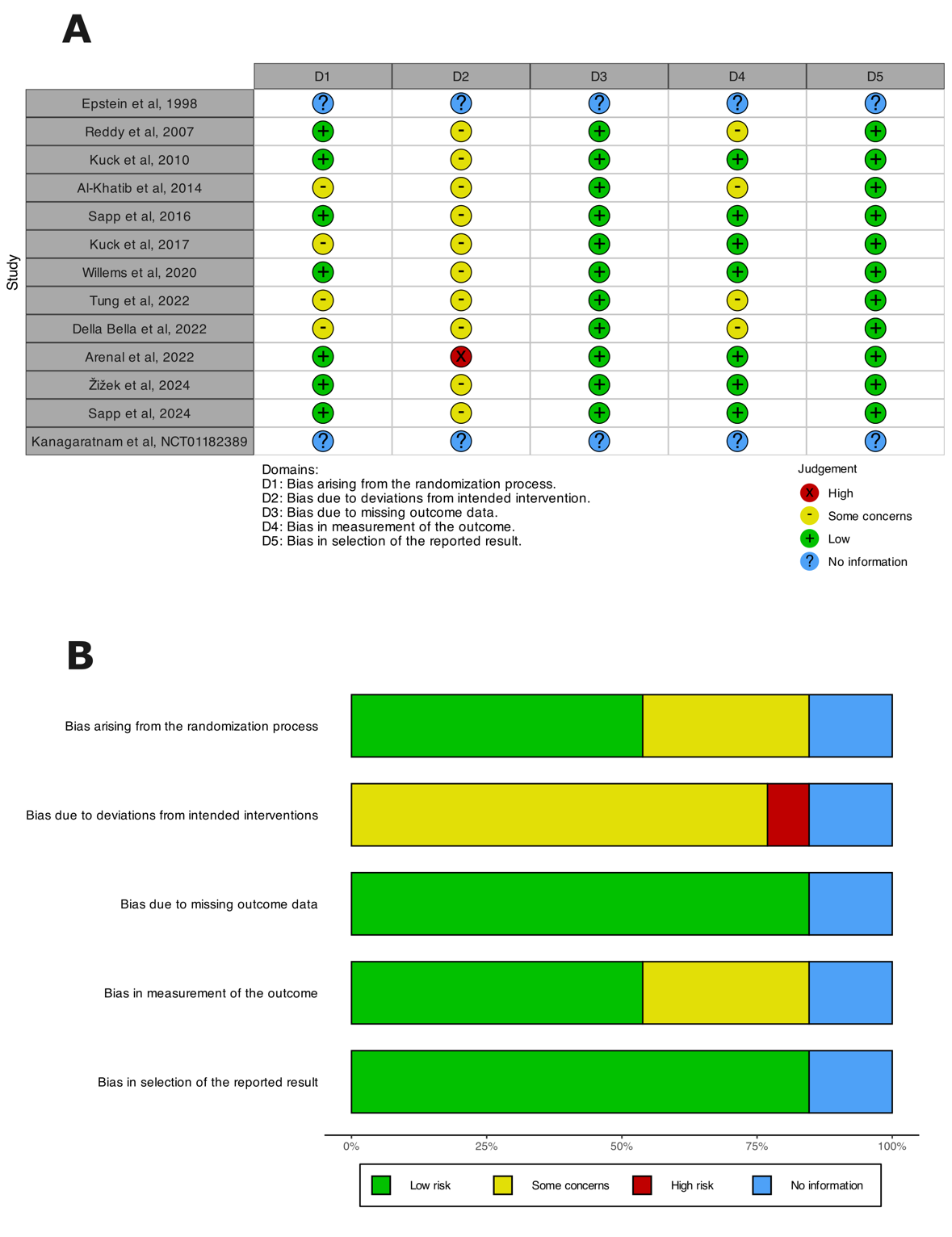


**Figure S-2**. Funnel plot for all-cause mortality


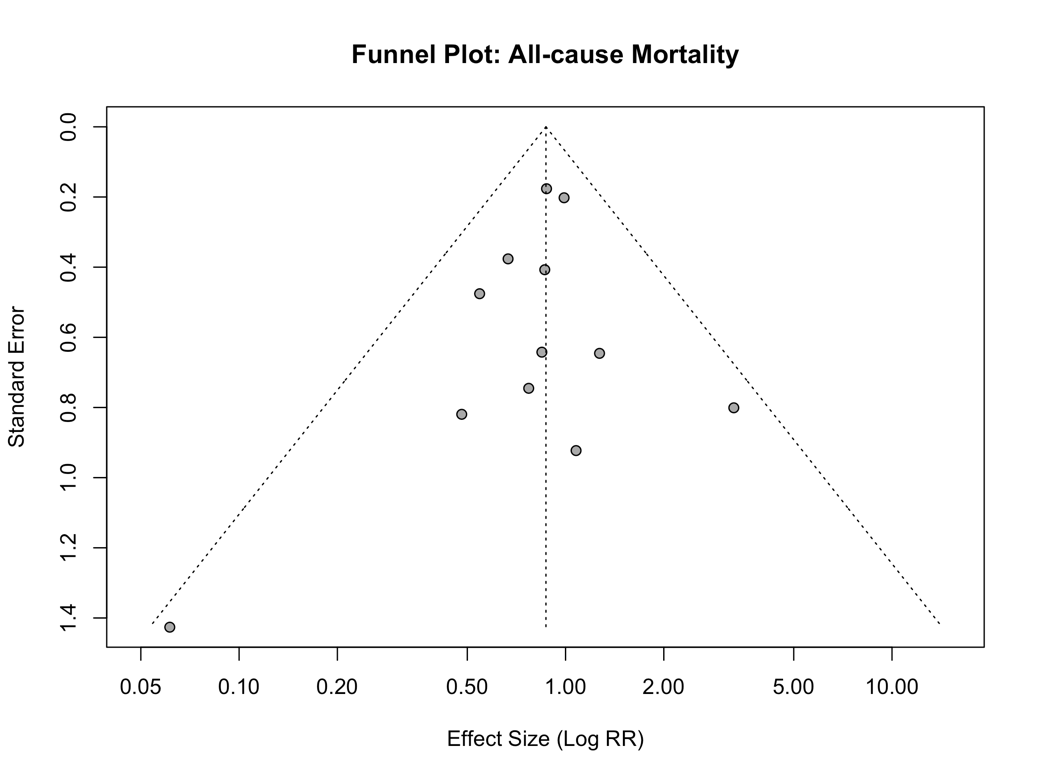


**Figure S-3:** Reconstructed all-cause mortality survival curves for each trial

| **BERLIN-VT**  **DOI:** 10.1161/CIRCULATIONAHA.119.043400 | **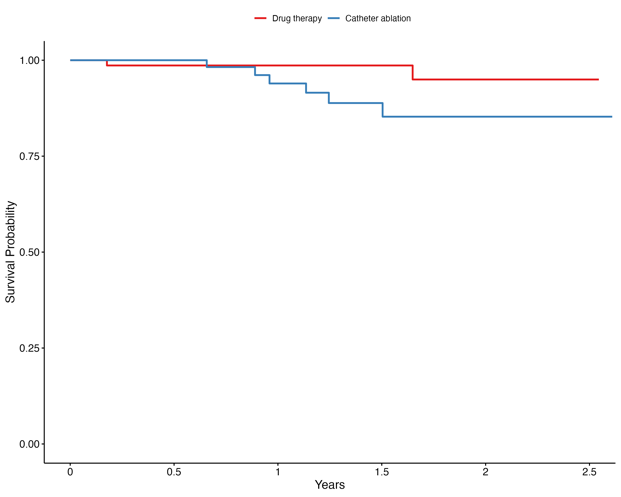** |
| --- | --- |
| **PARTITA**  **DOI:** 10.1161/CIRCULATIONAHA.122.059598 | **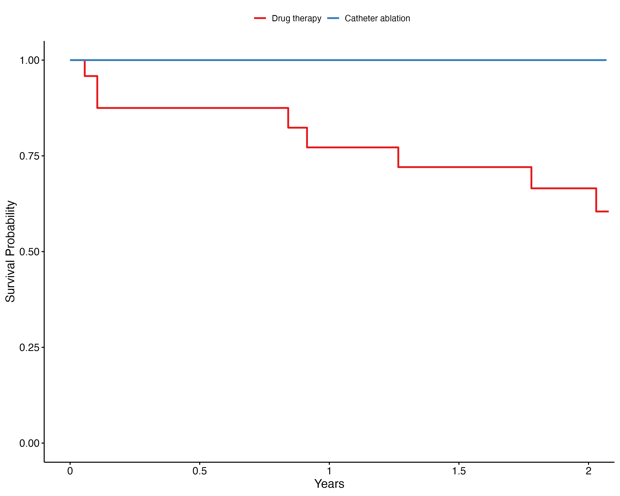** |
| **PAUSE-SCD**  **DOI:** 10.1161/CIRCULATIONAHA.122.060039 | **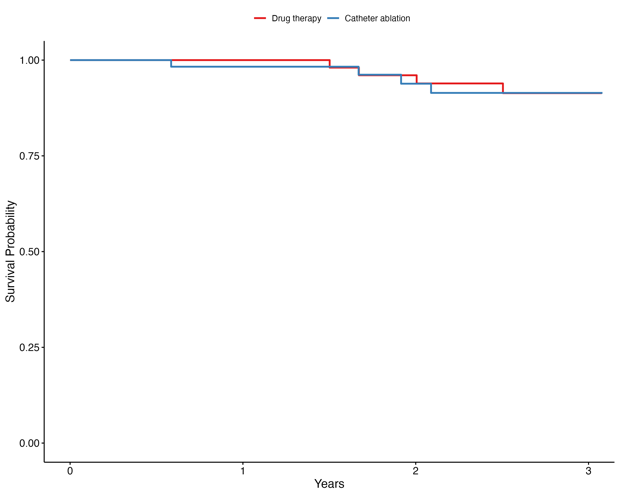** |
| **SMASH-VT**  **DOI:** 10.1056/NEJMoa065457 | **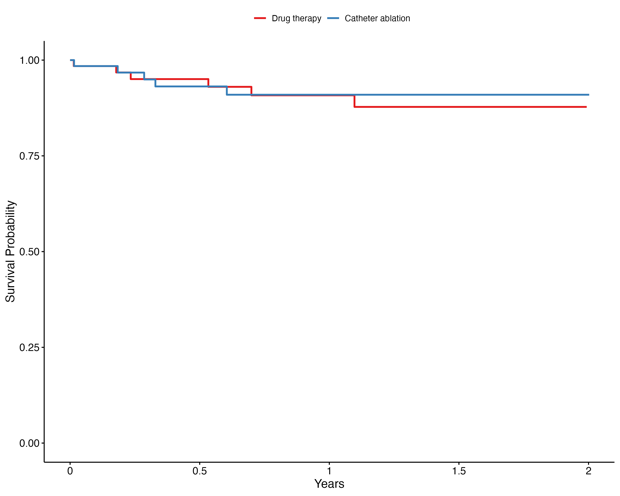** |
| **VANISH**  **DOI:** 10.1056/NEJMoa1513614 | **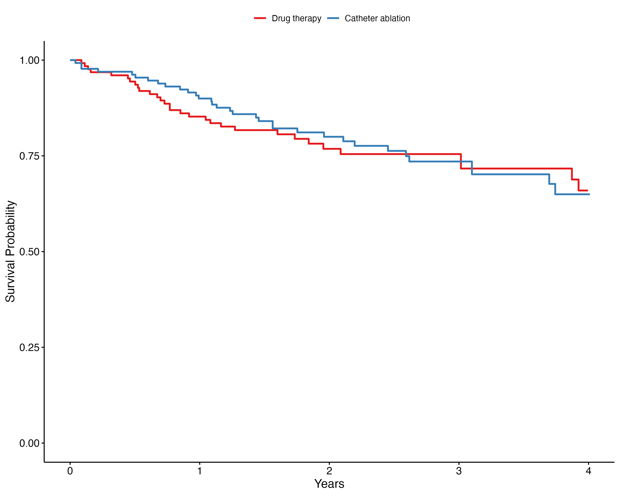** |
| **VANISH-2**  **DOI:** 10.1056/NEJMoa2409501 | **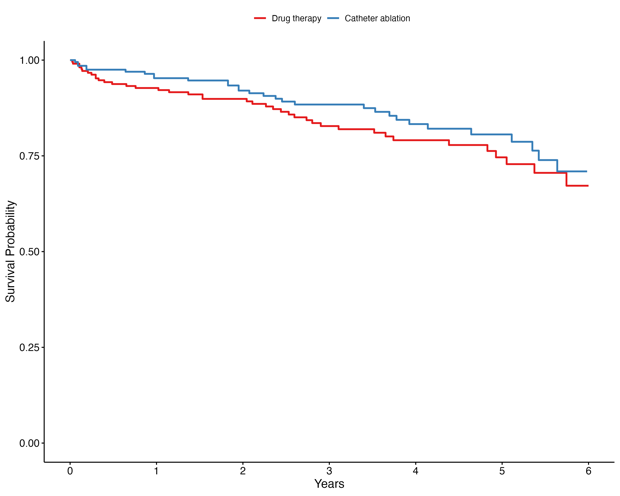** |

**Figure S-4**. Schoenfeld residual for individual patient data survival analysis


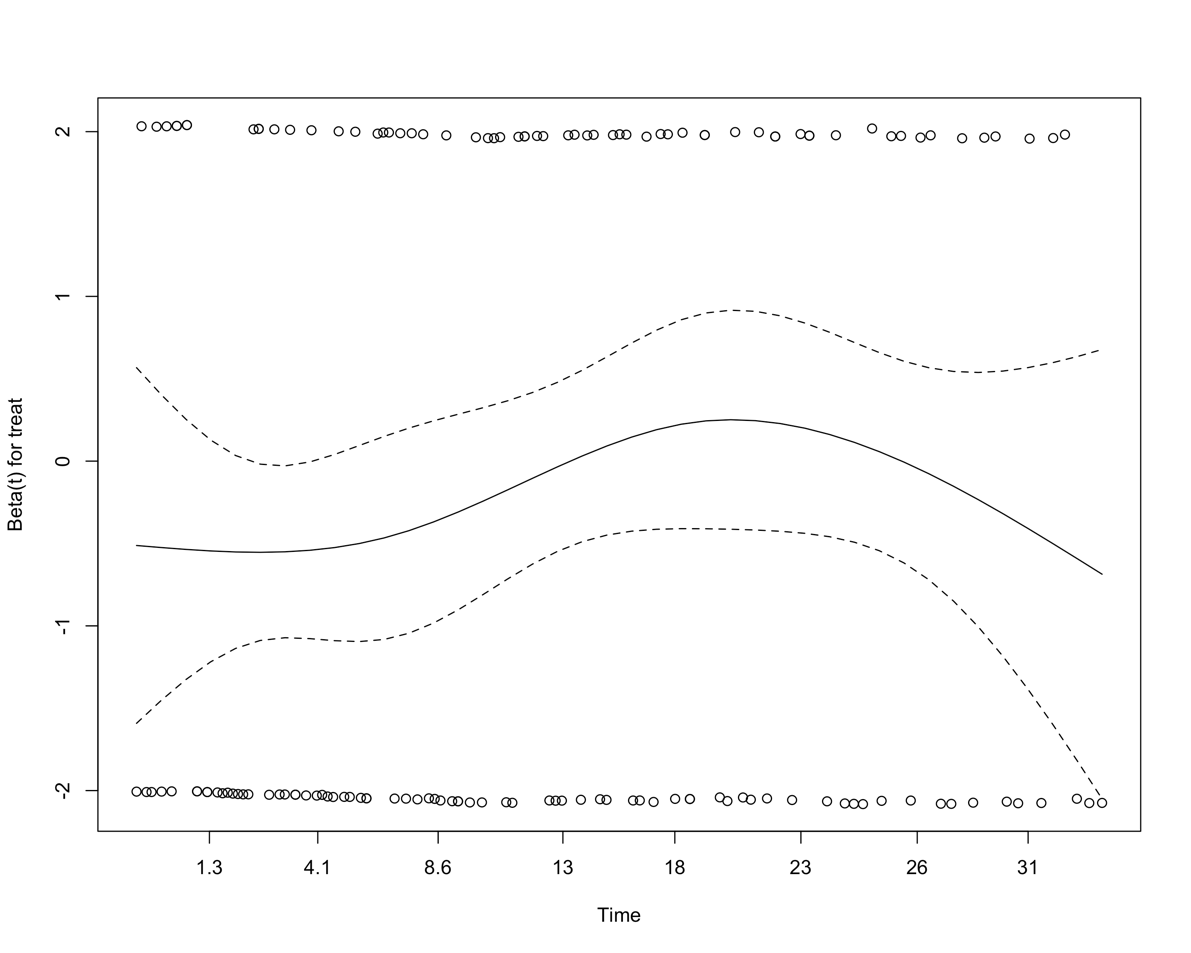


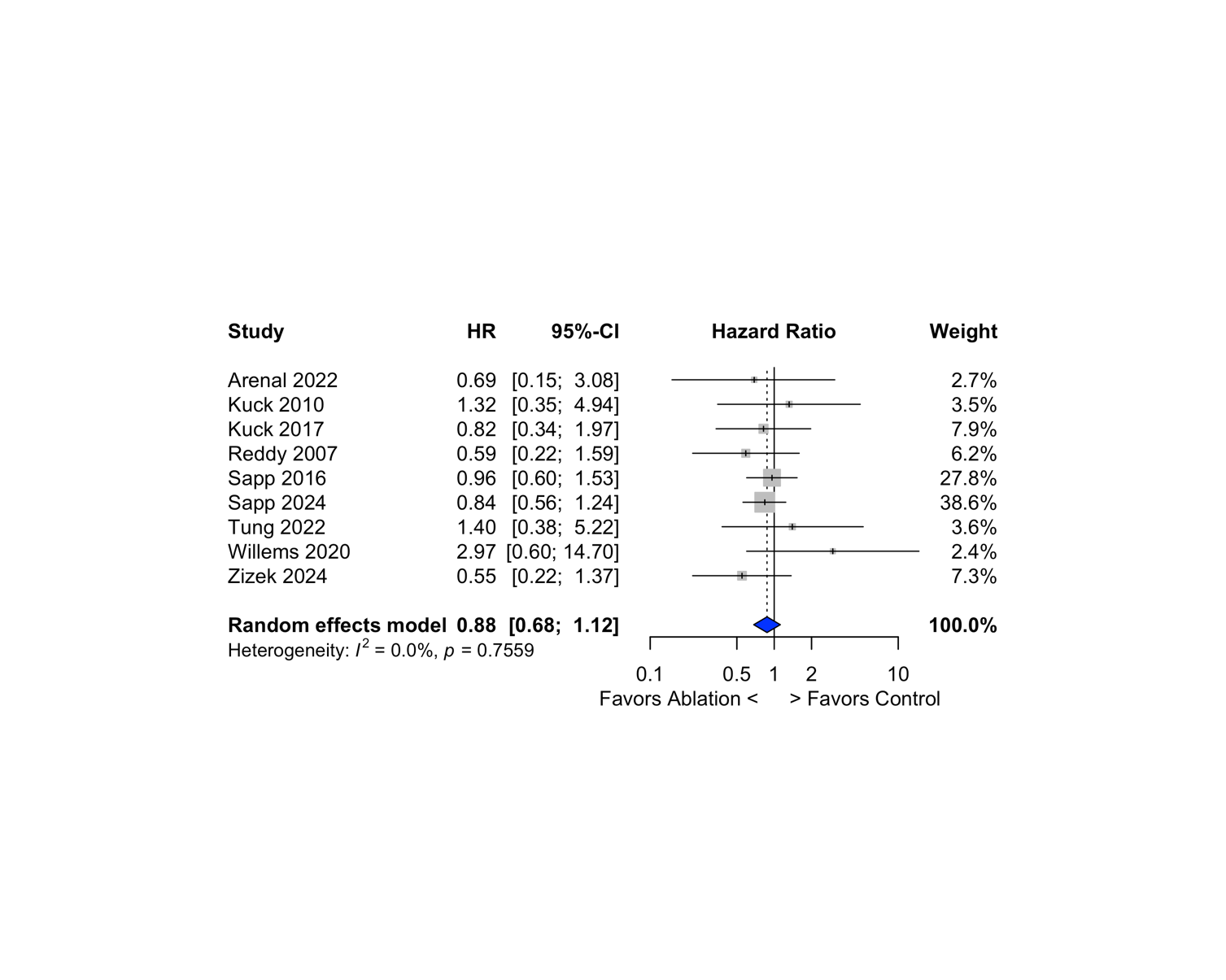
**Figure S-5**. Forest plot of pooled all-cause mortality hazard ratio

**Figure S-6**. Reconstructed all-cause mortality survival curves for individual patient data of ischemic cardiomyopathy comparing catheter ablation vs drug therapy


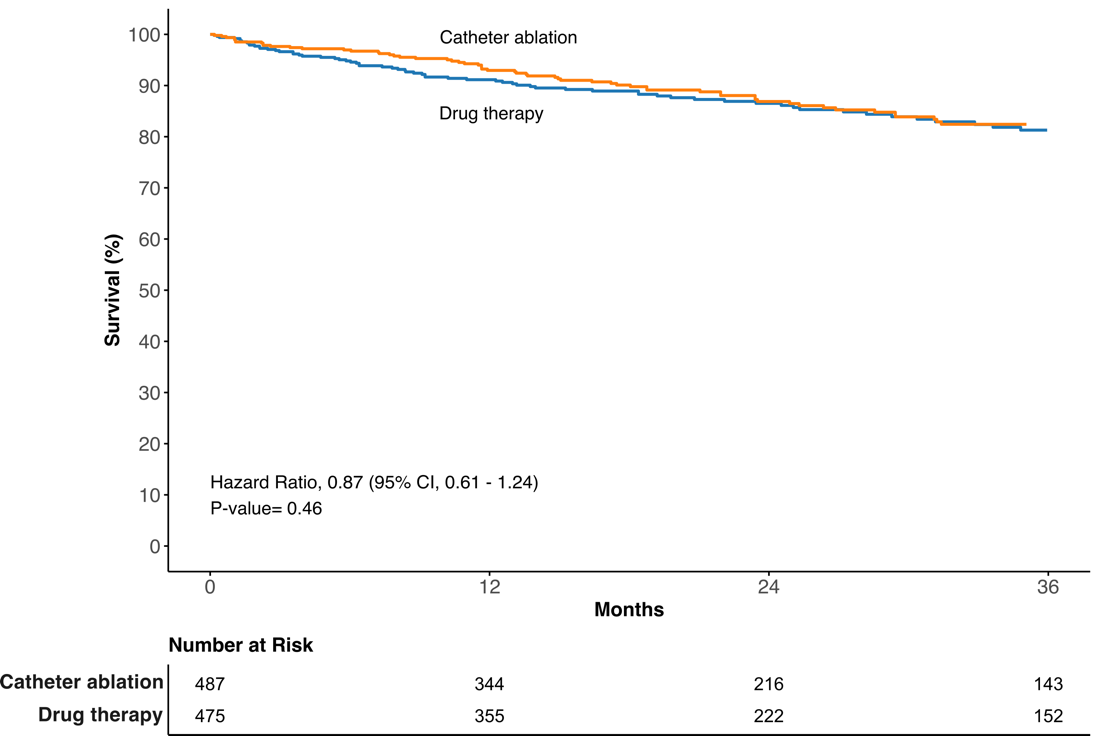


**Figure S-7**. Funnel plot for VT recurrence


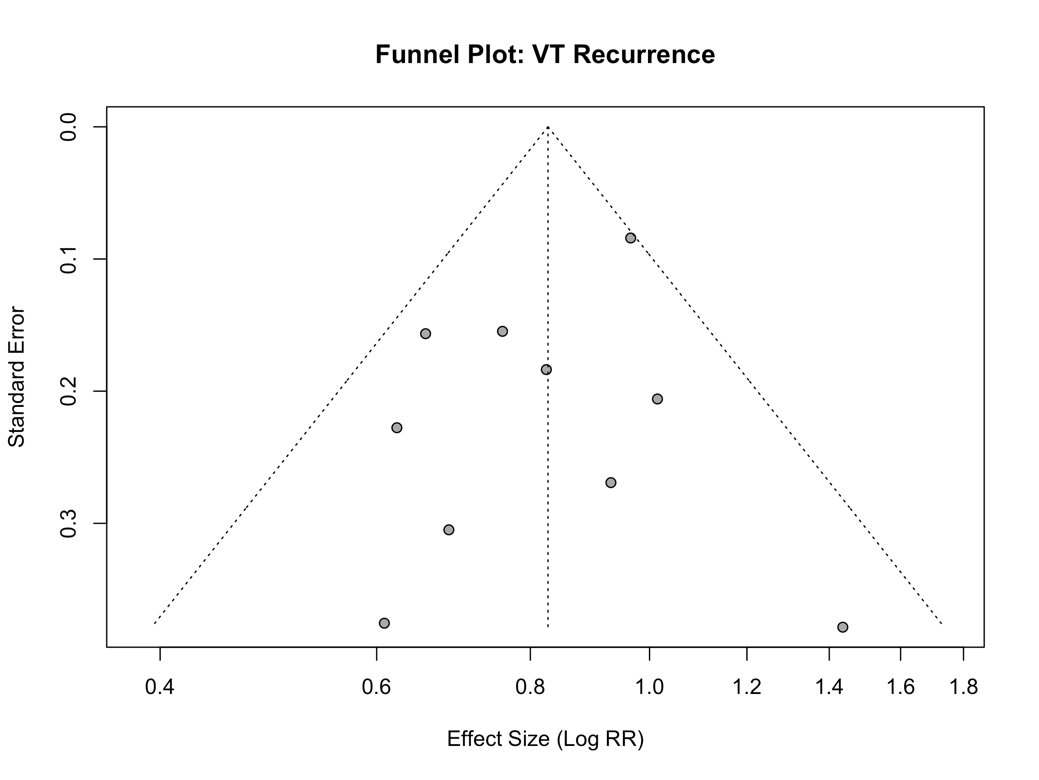


**
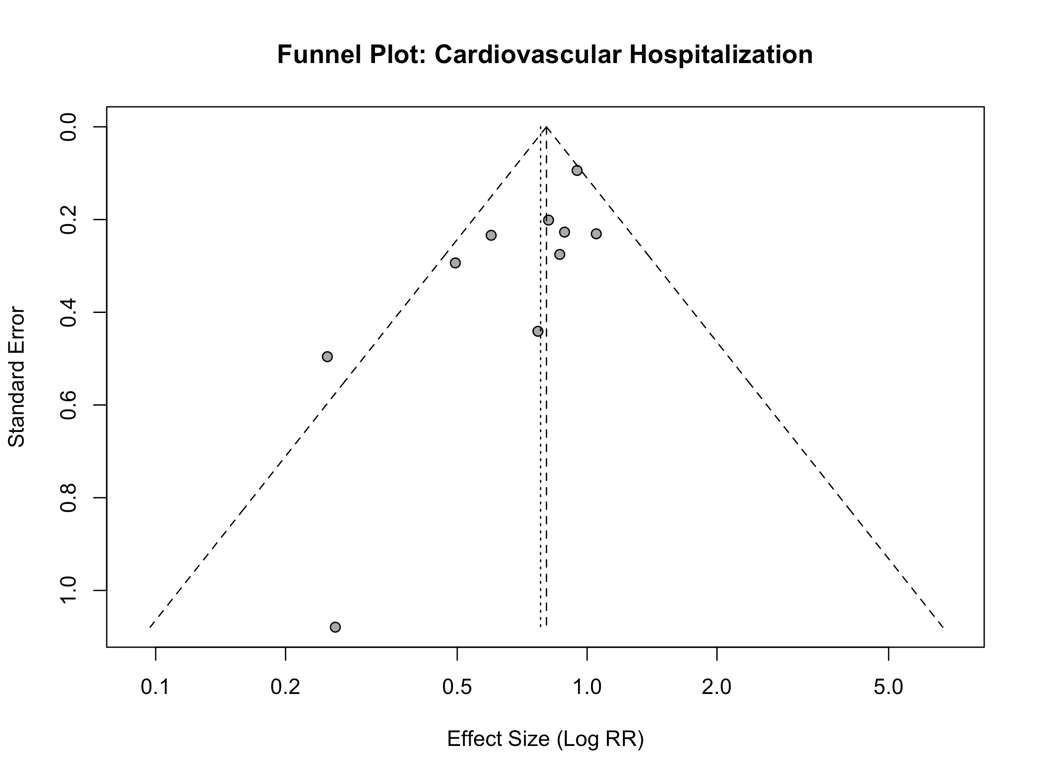
Figure S-8**. Funnel plot for cardiovascular hospitalization

**Figure S-9**. Forest plot of appropriate ICD shock


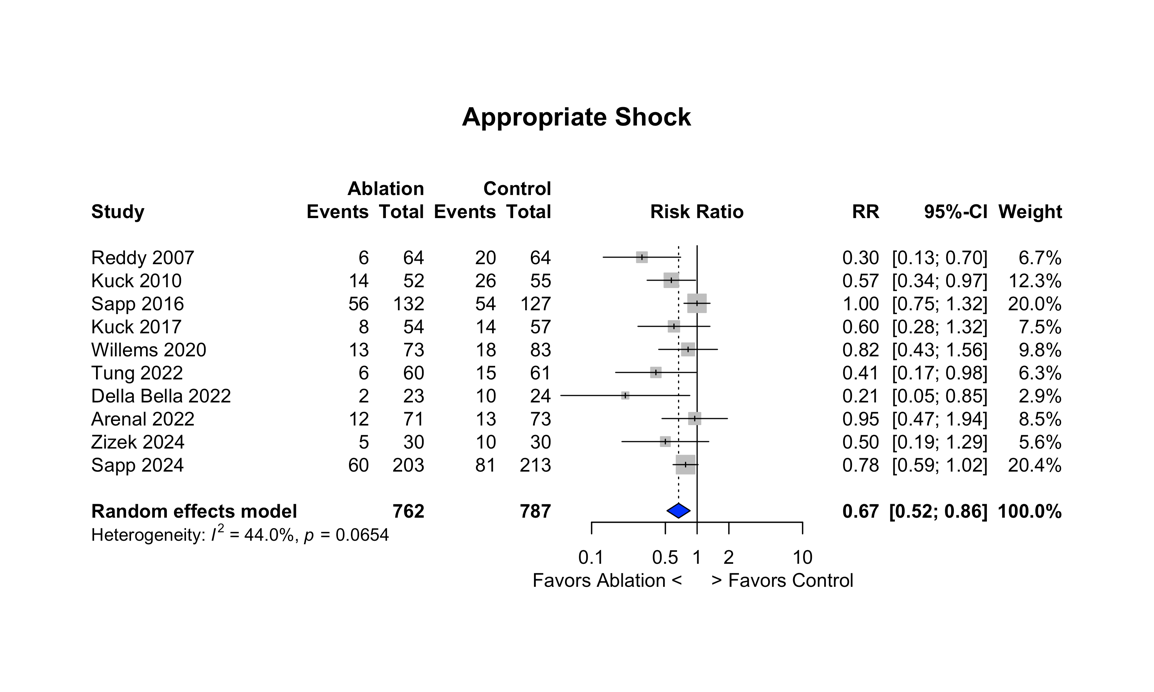


Abbreviation: VT, Ventricular Tachycardia; CI, confidence interval; RR, Risk Ratio.

**Figure S-10.** Leave-one-out sensitivity analysis for the pairwise meta-analysis

**Figure S-11.** Leave-one-out sensitivity analysis for the trial-level time-to-event analysis of all-cause mortality


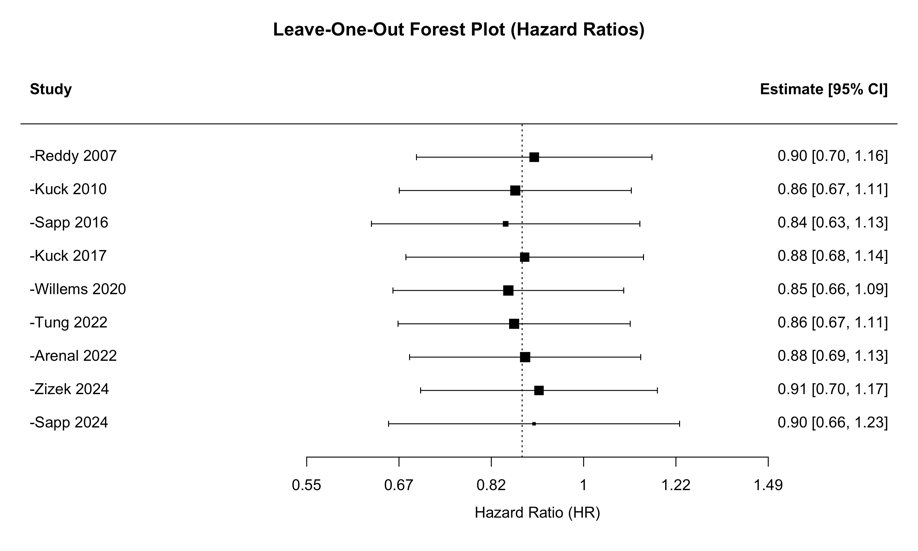

Supplement: oeaf171_Supplementary_Data [file oeaf171_supplementary_data.docx]
